# Incompatible Coulomb hamiltonian extensions

**DOI:** 10.1038/s41598-020-62144-2

**Published:** 2020-04-29

**Authors:** G. Abramovici

**Affiliations:** Université Paris-Saclay, CNRS, Laboratoire de Physique des Solides, 91405 Orsay, France

**Keywords:** Electronic properties and materials, Quantum mechanics

## Abstract

We revisit the resolution of the one-dimensional Schrödinger hamiltonian with a Coulomb *λ*/|*x*| potential. We examine among its self-adjoint extensions those which are compatible with physical conservation laws. In the one-dimensional semi-infinite case, we show that they are classified on a *U*(1) circle in the attractive case and on ${\boldsymbol{(}}{\mathbb{R}},{\boldsymbol{+}}{\boldsymbol{\infty }}{\boldsymbol{)}}$ in the repulsive one. In the one-dimensional infinite case, we find a specific and original classification by studying the continuity of eigenfunctions. In all cases, different extensions are incompatible one with the other. For an actual experiment with an attractive potential, the bound spectrum can be used to discriminate which extension is the correct one.

## Introduction

The Coulomb problem addresses the non-relativistic Schrödinger equation with a 3-dimensional Coulomb potential, restricted to one dimension; it has inspired a vast corpus of scientific literature for the last seventy years^1–11^. Some results have been much debated. Mathematical aspects are now fully understood, but physical ones want for more elaborated and robust interpretation, which we provide in details here.

In this article, we study the Coulomb potential, either restricted to a semi-infinite line, or else to a full infinite line. We will formally write the corresponding hamiltonian *H* = −*d*^2^/*dx*^2^ + *V* in dimensionless units and ${\mathbb{D}}$ will represent the domain on which wavefunctions are defined, so the first case corresponds to ${\mathbb{D}}={{\mathbb{R}}}_{+}^{\ast }$, while the second to ${\mathbb{D}}={\mathbb{R}}$. When necessary, we will write $H({\mathbb{D}})$ instead of *H*. One may note that the Schrödinger equation for ${\mathbb{D}}={{\mathbb{R}}}_{+}^{\ast }$ is equivalent, through a simple mapping, to the radial one for ${\mathbb{D}}={{\mathbb{R}}}^{3}$ in 3-dimension with zero orbital momentum, *L* = 0.

This work lies at the frontier between physics and mathematics, because Coulomb hamiltonians $H({{\mathbb{R}}}_{+}^{\ast })$ and $H({\mathbb{R}})$, although defined on a physical basis, reveal **non self-adjoint**. In such a case, one usually needs to study the self-adjoint **extensions** *K* of the hamiltonian. But, in this very case, the situation is even worse, because *H* is not even **symmetric**^6,11^ (that is, one can find two states *φ* and *χ* such that $\left\langle \varphi \right|H\left|\chi \right\rangle \ne \overline{\left\langle \chi \right|H\left|\varphi \right\rangle }$). In such a situation, one must **restrict** the Hilbert space on which eigenstates are defined, in order to get a symmetric operator, the self-adjoint extensions *K* of which are well-defined. We call ${\mathscr{L}}$ this restricted Hilbert space.

When the self-adjoint extension of an operator is unique, these mathematical manipulations are transparent because the spectral theorem applies, so the action of the operator is defined unambiguously on any function of ${\mathscr{L}}$. This is the case for almost all standard hamiltonians found in scientific literature, which are moreover generally well defined without any restriction (that is ${\mathscr{L}}={L}^{2}({\mathbb{D}})$), so one does not need to care about all these mathematical subtleties.

However, $H\left({{\mathbb{R}}}_{+}^{\ast }\right)$ and $H({\mathbb{R}})$ belong to the class of operators, which admit **several** self-adjoint extensions. Each extension is **incompatible** with the other, so one must **choose** only one extension at a time, where to define a complete set of eigenstates. From a physical point of view, the interpretation of the operator action on a wavefunction is ambiguous, since its definition **depends** on the extension which is chosen. *Deficiency coefficients* are defined, which indicate the number of degrees of freedom, for this choice. For $H({{\mathbb{R}}}_{+}^{\ast })$, authors have found^12–15^ one continuous degree of freedom.

## Motivation

The interest of the Coulomb problem lies in its unusual properties: the fact that hamiltonian $H({{\mathbb{R}}}_{+}^{\ast })$ and $H({\mathbb{R}})$ are not self-adjoint and not even symmetric, so that one must construct maximal restrictions ${\mathscr{L}}$ and study their self-adjoint extensions *K*. Our aim is to find a **physical** interpretation of these extensions, in order to identify those which are compatible with standard physical laws and those which are not.

The boundary triples theory, which is proved for the Coulomb problem^12^, establishes that any eigenfunction *ψ* of *K* is an eigenfunction of *H* with specific **boundary** conditions. This result, to which we will refer as the *boundary triples theorem*, provides a physical interpretation of all the self-adjoint extensions to be found. We will also benefit of all previous classifications of these extensions^12–15^ and repeat some of these calculations, taking into account physical considerations.

In what concerns the semi-infinite line, all self-adjoint extensions of $H({{\mathbb{R}}}_{+}^{\ast })$ reveal compatible with physical conservation laws, so the main contribution of this study on $H({{\mathbb{R}}}_{+}^{\ast })$ consists mainly in a more physical and pedagogical way to construct them. However, we provide an original description of the space parameter of these extensions, which is topologically equivalent to *U*(1) in the attractive case and to $({\mathbb{R}},\infty )$ in the repulsive one.

On the contrary, self-adjoint extensions of $H({\mathbb{R}})$ are not all compatible with physical conservation laws. Indeed, their study brings a specific difficulty: the connection of the solution defined on ${{\mathbb{R}}}_{+}^{\ast }$ with that defined on ${{\mathbb{R}}}_{-}^{\ast }$, since the continuity of eigenfunctions at *x* = 0 is not guaranteed. This has been very debated and we propose an original connection process, which is founded on physical conservation laws and gives **new**, although compatible, results.

Altogether, we prove a new classification of the self-adjoint extensions of $H({\mathbb{R}})$, **excluding** those which are not compatible with physical conservation laws. Accordingly, this classification maps on a space of extension parameter, which is **reduced** compared to that of previous classifications^15^, but the deficiency coefficient remains equal to 2. The parameter space of our classification is the product of a one-dimensional closed line by a phase similar to a gauge degree of freedom.

In what concerns the 3-dimension space, in spite of the mapping between its Schrödinger equation with that of $H({{\mathbb{R}}}_{+}^{\ast })$, the corresponding classifications of self-adjoint extensions are different (see however Appendix in Supplementary Information), since the deficiency coefficient of $H({{\mathbb{R}}}^{3})$ is zero^12^, that is $H({{\mathbb{R}}}^{3})$ is self-adjoint, when defined in ${L}^{2}({{\mathbb{R}}}^{3})$.

The present article is organized as follows: we will first focus on the ${\mathbb{D}}={{\mathbb{R}}}_{+}^{\ast }$ case and classify all self-adjoint extensions of $H({{\mathbb{R}}}_{+}^{\ast })$, both for an attractive potential or a repulsive one. In particular, we define and exhibit the Dirichlet or Neumann extensions. Then, we study in details the continuation problem in the ${\mathbb{D}}={\mathbb{R}}$ case. Next, we study physical applications of ${\mathbb{D}}={{\mathbb{R}}}^{3}$, ${\mathbb{D}}={\mathbb{R}}$ and ${\mathbb{D}}={{\mathbb{R}}}_{+}^{\ast }$ cases. In a fifth part, we examine the spectral theorem. In the next one, we exhibit the extension parameter spaces. Finally, we will review the highlights of this work on the Coulomb problem. Some notations and terms are given afterwards in Table 1.

**Table 1:** Notations and terminology.

*ℜ*/*ℑ*	real/imaginary part of a complex number
i	the imaginary number. Its conjugate reads ${}^{}\bar{i}=-{\mathbb{i}}$
${\mathbb{D}}$	generic physical space
${\mathbb{R}}$	set of real numbers
${{\mathbb{R}}}_{+}^{\ast }$ / ${\mathbb{N}}$	set of positive real/integer numbers
E^*^	the set *E* excluding 0 (for any set *E*)
*H*	hamiltonian
**simple**	without degeneracy
*η*	adimensional Coulomb parameter
Rydberg states	eigenstates corresponding to $-\eta \in {\mathbb{N}}$
non Rydberg states	eigenstates corresponding to $-\eta \notin {\mathbb{N}}$
${L}^{1}({\mathbb{D}})$	set of Lebesgue integrable functions defined in ${\mathbb{D}}$
${L}^{2}({\mathbb{D}})$	set of Lebesgue square integrable functions defined in ${\mathbb{D}}$
*L*_*n*_	Laguerre polynomial
${\mathscr{D}}$	generic domain where eigenstates are defined **for a given self-adjoint extension** not to be confused with boundary conditions in real space, applied to $H({\mathbb{D}})$
${\mathscr{S}}$	generic set of **negative** eigenvalues **for a given self-adjoint extension** (we call it spectrum instead of discrete spectrum)
${\mathscr{B}}$	generic set of **bound** eigenstates **for a given self-adjoint extension**
${\mathscr{F}}$	generic set of **free** eigenstates **for a given self-adjoint extension**
*ω*	parameter which classifies the self-adjoint extensions in the semi-infinite real line case
*ϖ* = (*ω*, *θ*)	parameter which classifies the self-adjoint extensions in the real line case

## Self-adjoint extensions in the ${{\mathbb{R}}}_{+}^{\ast }$ case

Operator $H({{\mathbb{R}}}_{+}^{\ast })$ is unbound and can not be defined on ${L}^{2}({{\mathbb{R}}}_{+}^{\ast })$, the Hilbert space of square-integrable functions. Eigenfunctions *ϕ*_*e*_ obey equation

1 $$-\frac{{d}^{2}{\phi }_{e}}{d{x}^{2}}(x)+\frac{\lambda }{x}{\phi }_{e}(x)=e\,{\phi }_{e}(x)\quad \forall x > 0$$

where we have multiplied Schrödinger equation by 2*m*/*ℏ*^2^, so *e* is the reduced energy corresponding to *E* = *ℏ*^2^*e*/(2*m*); we define $\lambda \equiv \frac{2mqq{\prime} }{4\pi {\varepsilon }_{o}{\hslash }^{2}}$, *m* is the mass of the particle, *ε*_o_ vacuum permittivity, *ℏ* the reduced Planck constant and *q*, $q{\prime}$ the electric charges. For *e* > 0 (free states of positive energy), the solutions of (1) read

$${\Psi }_{k}(x)={\alpha }_{k}{F}_{\eta }(kx)+{\beta }_{k}{G}_{\eta }(kx),\quad \mathrm{with}\,\mathrm{momentum}\,k\equiv \sqrt{e},\,\eta \equiv \lambda /(2k)\,{\rm{a}}{\rm{n}}{\rm{d}}$$

2 $$\begin{array}{ccc}{F}_{\eta }(u) & \equiv  & {C}_{\eta }u{{\rm{e}}}^{-{\mathbb{i}}u}M(1-{\mathbb{i}}\eta ,2,2{\mathbb{i}}u),\\ {G}_{\eta }(u) & \equiv  & {\rm{\Re }}\left(2\eta \frac{u{{\rm{e}}}^{-{\mathbb{i}}u}\Gamma (-{\mathbb{i}}\eta )}{{C}_{\eta }}U(1-{\mathbb{i}}\eta ,2,2{\mathbb{i}}u)\right),\\ {\rm{w}}{\rm{i}}{\rm{t}}{\rm{h}}\,{C}_{\eta } & \equiv  & {{\rm{e}}}^{-\frac{\pi \eta }{2}}\sqrt{\frac{\pi \eta }{\sinh (\pi \eta )}}.\end{array}$$

Here, Γ is the gamma function, *M* the regular confluent hypergeometric function and *U* the logarithmic confluent hypergeometric function^16^. Both *F*_*η*_ and *G*_*η*_ are continuous and bounded, see ref. ^11^ for asymptotic behavior and other properties. The case *e* = 0 extends this case when the potential is attractive, see section ‘Solutions of zero energies’.

For *e* < 0 (bound states of negative energy), the solutions of (1) read

$${\varphi }_{k}(x)={\mu }_{k}\,{f}_{\eta }(kx)+{\nu }_{k}{g}_{\eta }(kx),\quad \mathrm{with}\,\mathrm{momentum}\,k\equiv \sqrt{-{\rm{e}}},\,\eta \equiv \lambda /(2k)\,{\rm{a}}{\rm{n}}{\rm{d}}$$

3 $$\begin{array}{ccc}{f}_{\eta }(u) & \equiv  & 2{D}_{\eta }u{{\rm{e}}}^{-u}U(1+\eta ,2,2u),\\ {g}_{\eta }(u) & \equiv  & 2\sqrt{|\,\lambda \,|}u{{\rm{e}}}^{-u}M(1+\eta ,2,2u),\\ {\rm{w}}{\rm{i}}{\rm{t}}{\rm{h}}\,{D}_{\eta } & \equiv  & \frac{|\Gamma (1+\eta )|\sqrt{|\,\lambda \,|}}{\sqrt{1-2\eta +2{\eta }^{2}\,{\psi }_{{\rm{d}}{\rm{i}}{\rm{g}}}^{{\rm{{\prime} }}}(1+\eta )}}.\end{array}$$

Here, *ψ*_dig_ is the digamma function. One finds ${f}_{\eta }\in {L}^{1}({{\mathbb{R}}}_{+}^{\ast })$ $\bigcap \,{L}^{2}({{\mathbb{R}}}_{+}^{\ast })\,\bigcap \,{C}^{\infty }({{\mathbb{R}}}_{+}^{\ast })$ while ${g}_{\eta }\in {C}^{\infty }({{\mathbb{R}}}_{+}^{\ast })$ and diverges as *u* →∞. We have chosen ∥*f*_*η*_∥_2_ = 1 in ${L}^{2}({{\mathbb{R}}}_{+}^{\ast })$.

For *λ* < 0 so $qq{\prime}  < 0$ and the potential is attractive, the spectrum of any self-adjoint extension will reveal infinite and discrete. As we shall find, all solutions corresponding to *η* = −*n*, with $n\in {{\mathbb{N}}}^{\ast }$, belong to the same extension and read ${f}_{\eta }(u)=-u{{\rm{e}}}^{-u}{L}_{n}^{{\prime} }(2u)\sqrt{-2\lambda }\,{n}^{-3/2}$, the standard Rydberg solution, with *L*_*n*_ the Laguerre polynomial. They obey Dirichlet condition *f*_−*n*_(0) = 0. On the other hand, for $-\eta \notin {{\mathbb{N}}}^{\ast }$, *f*_*η*_(0) ≠ 0, see ref. ^11^ for more details. We will call *Rydberg* states, those following *η* = −*n* with $n\in {{\mathbb{N}}}^{\ast }$, and *non Rydberg* states the others. Note that the definition of *g*_*η*_ must be changed into

$${g}_{\eta }(u)=-u{{\rm{e}}}^{-u}{L}_{n}^{{\rm{{\prime} }}}(2u)\sqrt{-2\lambda }\,{n}^{-3/2}$$

since, in that very case *η* = −*n*, *u* e^−*u*^*M*(1 −*n*, 2, 2*u*) is proportional to *u* e^−*u*^*U*(1 −*n*, 2, 2*u*).

For *λ* > 0 so $qq{\prime}  > 0$ and the potential is repulsive, the spectrum of any self-adjoint extension will reveal discrete, with a unique bound state of strictly negative energy, but in a specific case that we will explain further on.

### Existence of self-adjoint extension

The existence of self-adjoint extensions for the Coulomb potential has been fully established in several references^12–15^ and needs not to be discussed here again. Indeed, the deficiency coefficients *m*_±_ are found equal to 1, although not explicitly calculated in ref. ^13^. We will construct all self-adjoint extensions as follows.

We will write ${H}_{\omega }({{\mathbb{R}}}_{+}^{\ast })$ the self-adjoint extensions of $H({{\mathbb{R}}}_{+}^{\ast })$, parametrized by *ω*, a symbolic index, the meaning of which will be explained later on. The boundary triples theorem implies that ${H}_{\omega }({{\mathbb{R}}}_{+}^{\ast })$ is the restriction of $H({{\mathbb{R}}}_{+}^{\ast })$ on some domain ${\mathscr{L}}$ of eigenfunctions, which we write ${\mathscr{L}}={{\mathscr{D}}}_{\omega }$. We will first construct all possible symmetric extensions of $H({{\mathbb{R}}}_{+}^{\ast })$ with different boundary conditions and find self-adjoint ones ${H}_{\omega }({{\mathbb{R}}}_{+}^{\ast })$ as **maximal** symmetric extensions^15^.

### Description of a self-adjoint extension

In this part, we consider the attractive case. Let *e*_*ω*_ < 0 be in the spectrum of ${H}_{\omega }({{\mathbb{R}}}_{+}^{\ast })$, that is ${\varphi }_{{k}_{\omega }}$, with momentum ${k}_{\omega }=\sqrt{-{e}_{\omega }}$, is an eigenfunction of ${H}_{\omega }({{\mathbb{R}}}_{+}^{\ast })$ and belongs to ${{\mathscr{D}}}_{\omega }$. There is such *e*_*ω*_, otherwise the spectrum of ${H}_{\omega }({{\mathbb{R}}}_{+}^{\ast })$ would be included in ${{\mathbb{R}}}_{+}$, which case we exclude later on. ${\varphi }_{{k}_{\omega }}$ is proportional to $x\mapsto {f}_{{\eta }_{\omega }}\,({k}_{\omega }x)$ (writing *η*_*ω*_ = *λ*/(2*k*_*ω*_)) because of (3); indeed, ${f}_{{\eta }_{\omega }}\,\in {L}^{2}({{\mathbb{R}}}_{+}^{\ast })$, so does ${\varphi }_{{k}_{\omega }}$ by definition, while ${g}_{{\eta }_{\omega }}$ diverges, letting ${\nu }_{{k}_{\omega }}=0$. The other factor reads then ${\mu }_{{k}_{\omega }}={{\rm{e}}}^{{\mathbb{i}}{\theta }_{\omega }}$, a constant phase factor which can be fixed arbitrarily.

One observes that not all functions *φ*_*k*_ belong to ${{\mathscr{D}}}_{\omega }$, because the scalar product $\langle {F}_{{\eta }_{1}}| {F}_{{\eta }_{2}}\rangle$,which we calculate in Appendix (see Supplementary Information), with arbitrary momenta *k*_*i*_ = *λ*/(2*η*_*i*_), is not always zero. Let us establish this result: we note *γ*_E_ the Euler constant and define function *g*_b_:

$${g}_{b}(x)\equiv {\psi }_{d{\mathbb{i}}g}(1+x)-ln\,| x| -\frac{1}{2x}+2{\gamma }_{E};$$

then, the scalar products reads

4 $$\langle {f}_{{\eta }_{1}}| \,{f}_{{\eta }_{2}}\rangle =\frac{{D}_{{\eta }_{1}}{D}_{{\eta }_{2}}\,{\lambda }^{2}}{{k}_{1}^{2}-{k}_{2}^{2}}\times \frac{{g}_{b}({\eta }_{2})-{g}_{b}({\eta }_{1})}{\Gamma (1+{\eta }_{1})\Gamma (1+{\eta }_{2})};$$

(this expression is valid when *η*_1_ → *η*_2_ and the limit is 1); therefore an operator admitting all such eigenfunctions would not be symmetric^6,11^. ■

Let ${{\mathscr{S}}}_{\omega }\equiv \left\{e\in {{\mathbb{R}}}_{-}^{\ast }/\langle {\varphi }_{{k}_{\omega }}| {\varphi }_{\sqrt{-e}}\rangle =0\right\}\bigcup \left\{{e}_{\omega }\right\}$. We will prove that the set of bound states of ${H}_{\omega }({{\mathbb{R}}}_{+}^{\ast })$ corresponds to functions generated by ${{\mathscr{B}}}_{\omega }\equiv \{{\varphi }_{k}\in {L}^{2}({{\mathbb{R}}}_{+}^{\ast })/{-k}^{2}\in {{\mathscr{S}}}_{\omega }\}$, so the spectrum of ${H}_{\omega }({{\mathbb{R}}}_{+}^{\ast })$ will exactly be ${{\mathscr{S}}}_{\omega }\,\bigcup \,{{\mathbb{R}}}_{+}$. Let us characterize ${{\mathscr{S}}}_{\omega }$. The condition $\langle {\varphi }_{{k}_{1}}| {\varphi }_{{k}_{2}}\rangle =0$ reduces to

5 $${g}_{b}({\eta }_{1})={g}_{b}({\eta }_{2})$$

so ${{\mathscr{S}}}_{\omega }=\left\{e/{g}_{b}\left(\frac{\lambda }{2\sqrt{-e}}\right)={g}_{b}({\eta }_{\omega })\right\}$. We study the zeros of *g*_b_(*η*) −*g*_b_(*η*_*ω*_) further on. (5) implies that any function *φ*_*k*_ orthogonal to ${\varphi }_{{k}_{\omega }}$ obeys *g*_b_(*η*) = *g*_b_(*η*_*ω*_) so all functions in ${{\mathscr{B}}}_{\omega }$ are either proportional or orthogonal to each other. By construction, ${{\mathscr{B}}}_{\omega }$ is maximal, because any function orthogonal to ${\varphi }_{{k}_{\omega }}$ belongs to it; there cannot be any other eigenfunction in ${{\mathscr{D}}}_{\omega }$ corresponding to a bound state, so $\{{\phi }_{e}\in {{\mathscr{D}}}_{\omega }/e\in {{\mathscr{S}}}_{\omega }\}\subseteq {{\mathscr{B}}}_{\omega }$. However, we cannot claim yet that this inclusion is an equality, because the scalar product of a bound state with a free one could be different from zero.

Let us discard this possibility and thus prove $\{{\phi }_{e}\in {{\mathscr{D}}}_{\omega }/e\in {{\mathscr{S}}}_{\omega }\}={{\mathscr{B}}}_{\omega }$. Let us examine free states. Let ${{\mathscr{F}}}_{\omega }$ be the set of functions *ϕ*_*e*_ = Ψ_*k*_, with *e* > 0 and momentum $k=\sqrt{e}$, such that

6 $$\left\langle {\varphi }_{{k}_{\omega }}| {\Psi }_{k}\right\rangle =0.$$

Each ${\phi }_{e}\in {{\mathscr{F}}}_{\omega }$ reads ${\phi }_{e}(x)={\alpha }_{\eta }^{\omega }{F}_{\eta }(k\,x)+{\beta }_{\eta }^{\omega }{G}_{\eta }(k\,x)$ using (2). Let us define *g*_f_:

$${g}_{f}(x)\equiv \Re \left({\psi }_{d{\mathbb{i}}g}(1+{\mathbb{i}}x)\right)+2{\gamma }_{E}-ln\,| x| ,$$

then the scalar products $\langle {f}_{{\eta }_{1}}| {F}_{{\eta }_{2}}\rangle$ and $\langle {F}_{{\eta }_{1}}| {G}_{{\eta }_{2}}\rangle$ calculated in Appendix (see Supplementary Information) read

7 $$\langle {f}_{{\eta }_{1}}| {F}_{{\eta }_{2}}\rangle =\frac{{D}_{{\eta }_{1}}{C}_{{\eta }_{2}}}{4{\eta }_{2}\Gamma (1+{\eta }_{1})}\times \frac{{\lambda }^{3/2}}{{k}_{1}^{2}+{k}_{2}^{2}};\,\langle {f}_{{\eta }_{1}}| {G}_{{\eta }_{2}}\rangle =\frac{{D}_{{\eta }_{1}}}{{C}_{{\eta }_{2}}}\frac{{g}_{b}({\eta }_{1})-{g}_{f}({\eta }_{2})}{2\Gamma (1+{\eta }_{1})}\times \frac{{\lambda }^{3/2}}{{k}_{1}^{2}+{k}_{2}^{2}}.$$

We define ${\zeta }_{\eta }^{\omega }\equiv {\alpha }_{\eta }^{\omega }/{\beta }_{\eta }^{\omega }$. For $-{\eta }_{1}\notin {{\mathbb{N}}}^{\ast }$ (non Rydberg states), using (6) with (7), one finds

8 $$\forall {e}_{1}=-\,{k}_{1}^{2}\in {{\mathscr{S}}}_{\omega }\qquad {\zeta }_{k}^{\omega }=\frac{2\eta }{{C}_{\eta }^{2}}\left({g}_{f}(\eta )-{g}_{b}({\eta }_{1})\right)=\frac{2\eta }{{C}_{\eta }^{2}}\left({g}_{f}(\eta )-{g}_{b}({\eta }_{\omega })\right)\,\mathrm{using}(5).$$

For $-{\eta }_{1}\in {{\mathbb{N}}}^{\ast }$ (Rydberg states), one finds $\langle {f}_{{\eta }_{1}}| {F}_{{\eta }_{2}}\rangle =0$ and $\langle {f}_{{\eta }_{1}}| {G}_{{\eta }_{2}}\rangle ={(-1)}^{{\eta }_{1}}\frac{\Gamma (-{\eta }_{1}){D}_{{\eta }_{1}}}{{C}_{{\eta }_{2}}}\frac{{\lambda }^{3/2}}{{k}_{1}^{2}+{k}_{2}^{2}}$ so one must choose ${\beta }_{{k}_{2}}=0$ and gets ${\zeta }_{{k}_{2}}=\infty$. (8) extends in this case, since *g*_b_(*η*_*ω*_) →∞ when *η*_*ω*_ → −*n* with $n\in {{\mathbb{N}}}^{\ast }$. (8) implies that Ψ_*k*_ is orthogonal to any function ${\varphi }_{{k}_{1}}\in {{\mathscr{B}}}_{\omega }$ as soon as it is orthogonal to ${\varphi }_{{k}_{\omega }}$. All free eigenfunctions of ${H}_{\omega }({{\mathbb{R}}}_{+}^{\ast })$ must belong to ${{\mathscr{F}}}_{\omega }$, so they respect (8); thus, they are all orthogonal to any ${\varphi }_{{k}_{1}}\in {{\mathscr{B}}}_{\omega }({{\mathbb{R}}}_{+}^{\ast })$; this ends our demonstration. ■

Conversely, all elements in ${{\mathscr{F}}}_{\omega }$ are eigenfunctions of ${H}_{\omega }({{\mathbb{R}}}_{+}^{\ast })$. In that purpose, let us establish the generalized orthonormality of all elements in ${{\mathscr{F}}}_{\omega }$. Let ${\phi }_{{e}_{1}}$ and ${\phi }_{{e}_{2}}$ be in ${{\mathscr{F}}}_{\omega }$, with *e*_1_ ≠ *e*_2_. The scalar products $\langle {F}_{{\eta }_{1}}| {F}_{{\eta }_{2}}\rangle$, $\langle {F}_{{\eta }_{1}}| {G}_{{\eta }_{2}}\rangle$, $\langle {G}_{{\eta }_{1}}| {F}_{{\eta }_{2}}\rangle$ and $\langle {G}_{{\eta }_{1}}| {G}_{{\eta }_{2}}\rangle$, calculated in Appendix (see Supplementary Information) read

9 $$\begin{array}{rcl}\langle {G}_{{\eta }_{1}}| {G}_{{\eta }_{2}}\rangle  & = & \frac{\lambda }{{C}_{{\eta }_{1}}{C}_{{\eta }_{2}}}\frac{{g}_{f}({\eta }_{2})-{g}_{f}({\eta }_{1})}{{k}_{1}^{2}-{k}_{2}^{2}}+\delta ({k}_{1}-{k}_{2});\\ \langle {F}_{{\eta }_{1}}| {F}_{{\eta }_{2}}\rangle  & = & \delta ({k}_{1}-{k}_{2});\\ \langle {F}_{{\eta }_{1}}| {G}_{{\eta }_{2}}\rangle  & = & \frac{\lambda {C}_{{\eta }_{1}}}{2{\eta }_{1}{C}_{{\eta }_{2}}}\frac{1}{{k}_{1}^{2}-{k}_{2}^{2}}.\end{array}$$

For *e*_1_ ≠ *e*_2_, we span the scalar product $\langle {\phi }_{{e}_{1}}| {\phi }_{{e}_{2}}\rangle$ using (2) and (8), which gives

$$\begin{array}{rcl}\langle {\phi }_{{e}_{1}}| {\phi }_{{e}_{2}}\rangle  & = & \bar{{\alpha }_{{\eta }_{1}}^{\omega }}{\alpha }_{{\eta }_{2}}^{\omega }\langle {F}_{{\eta }_{1}}| {F}_{{\eta }_{2}}\rangle +\bar{{\alpha }_{{\eta }_{1}}^{\omega }}{\beta }_{{\eta }_{2}}^{\omega }\langle {F}_{{\eta }_{1}}| {G}_{{\eta }_{2}}\rangle +\bar{{\beta }_{{\eta }_{1}}^{\omega }}{\alpha }_{{\eta }_{2}}^{\omega }\langle {G}_{{\eta }_{1}}| {F}_{{\eta }_{2}}\rangle +\bar{{\beta }_{{\eta }_{1}}^{\omega }}{\beta }_{{\eta }_{2}}^{\omega }\langle {G}_{{\eta }_{1}}| {G}_{{\eta }_{2}}\rangle \\  & = & \frac{{\eta }_{1}^{2}{\eta }_{2}^{2}}{\lambda {C}_{{\eta }_{1}}{C}_{{\eta }_{2}}}\frac{\bar{{\beta }_{{\eta }_{1}}^{\omega }}{\beta }_{{\eta }_{2}}^{\omega }}{{\eta }_{1}^{2}-{\eta }_{2}^{2}}(0-2{\eta }_{1}(\,{g}_{f}({\eta }_{1})-{g}_{b}({\eta }_{\omega }))\frac{2}{{\eta }_{1}}+\,2{\eta }_{2}(\,{g}_{f}({\eta }_{2})-{g}_{b}({\eta }_{\omega }))\frac{2}{{\eta }_{2}}-4(\,{g}_{f}({\eta }_{2})-{g}_{f}({\eta }_{1})))\\  &  & +\,(| {\alpha }_{{\eta }_{1}}^{\omega }{| }^{2}+| {\beta }_{{\eta }_{1}}^{\omega }{| }^{2})\delta ({k}_{1}-{k}_{2})\\  & = & \delta ({k}_{1}-{k}_{2}),\end{array}$$

where $$\bar{{\zeta }_{{\eta }_{1}}}={\zeta }_{{\eta }_{1}}$$ follows (8). ■

We have proved that all bound eigenfunctions of ${H}_{\omega }({{\mathbb{R}}}_{+}^{\ast })$ are in ${{\mathscr{B}}}_{\omega }$ while all free ones are in ${{\mathscr{F}}}_{\omega }$. Therefore, we get ${{\mathscr{D}}}_{\omega }={{\mathscr{B}}}_{\omega }\bigcup {{\mathscr{F}}}_{\omega }$. We define ${\widetilde{H}}_{\omega }$ the restriction of $H({{\mathbb{R}}}_{+}^{\ast })$ on ${{\mathscr{D}}}_{\omega }$. We will prove now that ${\widetilde{H}}_{\omega }$ is symmetric, that is $\langle ({\widetilde{H}}_{\omega }\psi )| \varphi \rangle =\langle \psi | ({\widetilde{H}}_{\omega }\varphi )\rangle$ for all $\psi ,\varphi \in {{\mathscr{D}}}_{\omega }$. Let (*e*_1_, *e*_2_) be such that $\psi ={\phi }_{{e}_{1}}$ and $\varphi ={\phi }_{{e}_{2}}$ (depending on whether *ψ* belongs to the free or the bound spectrum, either ${e}_{1}\in {{\mathbb{R}}}^{+}$ or ${e}_{1}\in {{\mathscr{S}}}_{\omega }$, and idem for *φ* with *e*_2_). One writes then

$$\left\langle ({\widetilde{H}}_{\omega }\psi )| \varphi \right\rangle -\left\langle \psi | ({\widetilde{H}}_{\omega }\varphi )\right\rangle =\mathop{\underbrace{\bar{{e}_{1}}}}\limits_{={e}_{1}}\left\langle \psi | \varphi \right\rangle -{e}_{2}\left\langle \psi | \varphi \right\rangle =({e}_{1}-{e}_{2})\left\langle \psi | \varphi \right\rangle =0$$

The last equality is proved by discussing whether *e*_1_ ≠ *e*_2_, so $\psi ={\phi }_{{e}_{1}}\perp {\phi }_{{e}_{2}}$ = *φ*, following all previous discussions, or else *e*_1_ = *e*_2_. ■

Let us prove that ${\widetilde{H}}_{\omega }$ is maximal ad absurdum. Since it is symmetric, it admits a self-adjoint extension *K*, which is defined on ${\mathscr{C}}\bigcup {{\mathscr{D}}}_{\omega }$, where ${\mathscr{C}}$ is some non empty space, by hypothesis. Let us write ${K}_{{\mathscr{C}}}$ the restriction of *K* on ${\mathscr{C}}$. Let $\{{\phi }_{i},i\in {\mathscr{I}}\}$ be a basis of ${{\mathscr{D}}}_{\omega }$, and $\{{\psi }_{j},j\in {\mathscr{J}}\}$ a basis of ${\mathscr{C}}$. One writes

$$K\left|{\phi }_{i}\right\rangle ={\widetilde{H}}_{\omega }\left|{\phi }_{i}\right\rangle =\sum _{k\in {\mathscr{I}}}{a}_{i}^{k}\left|{\phi }_{k}\right\rangle \qquad K\left|{\psi }_{j}\right\rangle =\sum _{k\in {\mathscr{I}}}{b}_{j}^{k}\left|{\phi }_{k}\right\rangle +\sum _{l\in {\mathscr{J}}}{c}_{j}^{l}\left|{\psi }_{l}\right\rangle .$$

Multiplying the first line by $\left\langle {\psi }_{j}\right|$ and the second by $\left\langle {\phi }_{{\mathbb{i}}}\right|$, one gets ${b}_{j}^{{\mathbb{i}}}=0$, so $\left\langle {\phi }_{{\mathbb{i}}}| {\psi }_{j}\right\rangle =0$ ∀*i*, *j*. *K* is symmetric, so $\left\langle {\psi }_{{\mathbb{i}}}\right|K\left|{\psi }_{j}\right\rangle =\overline{\left\langle {\psi }_{j}\right|K\left|{\psi }_{{\mathbb{i}}}\right\rangle }$, which implies ${c}_{{\mathbb{i}}}^{j}=\bar{{c}_{j}^{{\mathbb{i}}}}$ ∀*i*, *j*. Eventually, we have established that ${K}_{{\mathscr{C}}}$ is symmetric. From standard algebra^17^, there exists at least an eigenfunction ${\phi }_{0}\in {\mathscr{C}}$, and its eigenvalue *e*_0_ is real.

Applying the boundary triples theorem, the function *ϕ*_0_ is a solution of the differential equation $H({{\mathbb{R}}}_{+}^{\ast }){\phi }_{0}(x)={e}_{0}{\phi }_{0}(x)$, with particular boundary conditions. If *e*_0_ < 0, one finds immediately that ${\phi }_{0}\in {{\mathscr{B}}}_{\omega }$. If *e*_0_ ≥ 0, one must first write that $\left\langle {\varphi }_{k}| {\phi }_{0}\right\rangle =0$ for all *k* (*φ*_*k*_ are the elements of ${{\mathscr{B}}}_{\omega }$); following the previous construction, one eventually finds that ${\phi }_{0}\in {{\mathscr{F}}}_{\omega }$. We have proved ${\phi }_{0}\in {{\mathscr{D}}}_{\omega }$, which contradicts ${\phi }_{0}\in {\mathscr{C}}$, so the maximality of ${\widetilde{H}}_{\omega }$ is proved. ■

${\widetilde{H}}_{\omega }$ is symmetric and maximal, that is, it is a self-adjoint extension. Furthermore, ${\widetilde{H}}_{\omega }$ is simple. Concerning bound states, this results from the elimination of functions proportional to *g*_*η*_. Concerning free states, it follows (8). Now, let *ϕ*_*e*_ be any eigenfunction included in the domain of ${H}_{\omega }({{\mathbb{R}}}_{+}^{\ast })$. This domain includes ${\phi }_{{e}_{\omega }}$, so *ϕ*_*e*_ must be either orthogonal to ${\phi }_{{e}_{\omega }}$ or have eigenvalue *e*_*ω*_. In the first case, *ϕ*_*e*_ belongs to ${{\mathscr{D}}}_{\omega }$. In the second case, it is proportional to ${\phi }_{{e}_{\omega }}$ (still resulting from the elimination of functions proportional to ${g}_{{\eta }_{\omega }}$). This proves that the domain of ${H}_{\omega }({{\mathbb{R}}}_{+}^{\ast })$ is included in that of ${\widetilde{H}}_{\omega }$, so ${\widetilde{H}}_{\omega }$ is an extension of ${H}_{\omega }({{\mathbb{R}}}_{+}^{\ast })$. Self-adjoint extensions are maximal, so ${\widetilde{H}}_{\omega }={H}_{\omega }({{\mathbb{R}}}_{+}^{\ast })$, which is therefore completely determinate. ■

### Classification in the attractive case

**Figure 1:**
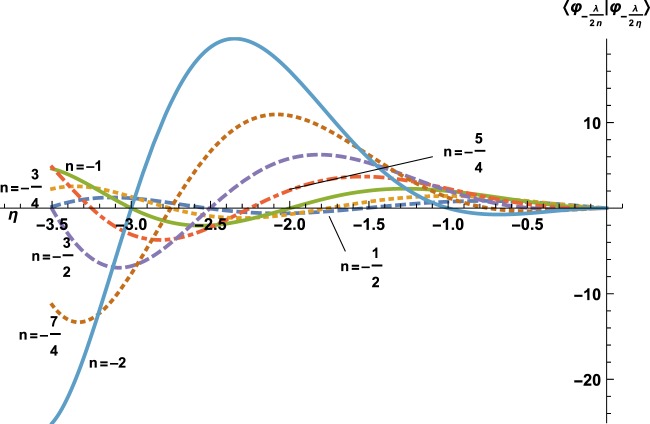
Here are the curves $\eta \mapsto \langle {\varphi }_{-\frac{\lambda }{2n}}| {\varphi }_{-\frac{\lambda }{2\eta }}\rangle$, for *n* = −1/2 (dashed line), *n* = −3/4 (dotted line), *n* = −1 (plain line), *n* = −5/4 (dot-dashed line), *n* = −3/2 (dashed line), *n* = −7/4 (dotted line) and *n* = −2 (plain line). The zeros of each curve read $\eta =\frac{\lambda }{2\sqrt{-e}}$ where $e\in {{\mathscr{S}}}_{\omega }$, with *ω* = −*g*_b_(*n*), as explained further on. The curves seem to form pairs corresponding to (*n*, *n* + 1), in particular, one could believe that each pair intersects on the *η*-axis (abscissa), but this is wrong, except for (*n*, *n* + 1) = ( −2, −1) which correspond to the same Rydberg set ${{\mathscr{S}}}_{\infty }$. All the other intersections are only close to zero, so that, indeed, ${{\mathscr{S}}}_{-{g}_{b}(n)}\ne {{\mathscr{S}}}_{-{g}_{b}(n+1)}$. *η* = *n* is missing, because $\langle {\varphi }_{-\frac{\lambda }{2n}}| {\varphi }_{-\frac{\lambda }{2n}}\rangle \ne 0$.

Set ${{\mathscr{S}}}_{\omega }$ contains the zeros of $\eta \mapsto {g}_{b}\left(-{\left(\frac{\lambda }{2\eta }\right)}^{2}\right)-{g}_{b}({e}_{\omega })$, which we represent for several values of *ω* in Fig. 1. To characterize each set ${{\mathscr{B}}}_{\omega }$, we follow the results in ref. ^12^ and define ${\widetilde{{\mathscr{B}}}}_{\omega }=\{{\varphi }_{k}\in {L}^{2}({{\mathbb{R}}}_{+}^{\ast })/\frac{\partial {\varphi }_{k}(x)}{| \,\lambda \,| \partial x}+{\varphi }_{k}(x)ln\,(| \,\lambda \,| x)=\omega {\varphi }_{k}(x)\}$. This condition differs from the more usual one $\frac{\partial \phi (k\,x)}{\partial x}=\omega \phi (k\,x)$. Another possible characterization is given in ref. ^13^. For a given number $\omega \in {\mathbb{R}}$, we define *η*_*ω*_ to be any solution of *g*_b_(*η*) = −*ω* (one can chose the highest *η*, as we will prove further on that this set has a maximum).

Let us prove ${{\mathscr{B}}}_{\omega }={\widetilde{{\mathscr{B}}}}_{\omega }$. First, we will show that two functions in ${\widetilde{{\mathscr{B}}}}_{\omega }$ are either proportional or orthogonal. One finds, for non Rydberg eigenfunctions ($-\eta \notin {{\mathbb{N}}}^{\ast }$),

$$\begin{array}{rl}\frac{\partial {f}_{\eta }\left(\frac{\lambda x}{2\eta }\right)}{\partial x}=\frac{\lambda }{2\eta }{f}_{\eta }^{{\prime} }\left(\frac{\lambda x}{2\eta }\right) & \mathrm{so}\,\mathop{\mathrm{lim}}\limits_{x\to 0}\frac{\partial {f}_{\eta }\left(\frac{\lambda x}{2\eta }\right)}{| \,\lambda \,| \partial x}+{f}_{\eta }\left(\frac{\lambda x}{2\eta }\right)ln\,(| \,\lambda \,| x)=-\frac{{g}_{b}(\eta ){D}_{\eta }}{\Gamma (1+\eta )}\\ \mathrm{while}\,\mathop{\mathrm{lim}}\limits_{x\to 0}{f}_{\eta }\left(\frac{\lambda x}{2\eta }\right)=\frac{{D}_{\eta }}{\Gamma (1+\eta )} & \mathrm{so}\,\mathop{\mathrm{lim}}\limits_{x\to 0}\frac{\frac{\partial {f}_{\eta }\left(\frac{\lambda x}{2\eta }\right)}{| \,\lambda \,| \partial x}+{f}_{\eta }\left(\frac{\lambda x}{2\eta }\right)ln\,(| \,\lambda \,| x)}{{f}_{\eta }\left(\frac{\lambda x}{2\eta }\right)}=-{g}_{b}(\eta ).\end{array}$$

Thus, it comes that all elements in ${\widetilde{{\mathscr{B}}}}_{\omega }$ verify *ω* = −*g*_b_(*η*), so, using (5), the proposition is proved, except for Rydberg states such that $-\eta \in {{\mathbb{N}}}^{\ast }$. For these, the last limit gives ∞. However, these eigenfunctions are well known and indeed orthogonal (see section ‘Dirichlet solutions’), so the result extends to this case immediately. Conversely, any index *η* corresponding to ${\varphi }_{k}\in {{\mathscr{B}}}_{\omega }$ verifies *g*_b_(*η*) = *g*_b_(*η*_*ω*_) = −*ω*. Eventually, this proves ${\widetilde{{\mathscr{B}}}}_{\omega }={{\mathscr{B}}}_{\omega }$. ■

Let’s define ${\widetilde{{\mathscr{F}}}}_{\omega }=\{{\Psi }_{k}\in {L}^{\infty }({{\mathbb{R}}}_{+}^{\ast })/\frac{\partial {\Psi }_{k}(x)}{| \,\lambda \,| \partial x}+{\Psi }_{k}(x)ln\,(| \,\lambda \,| x)=\omega {\Psi }_{k}(x)\}$. We will prove now that ${\widetilde{{\mathscr{F}}}}_{\omega }={{\mathscr{F}}}_{\omega }$. We first show ${\mathscr{F}}\subset {\widetilde{{\mathscr{F}}}}_{\omega }$. One finds

$$\begin{array}{rl}\mathop{\mathrm{lim}}\limits_{x\to 0}\mathop{\mathrm{lim}}\limits_{x\to 0}{F}_{\eta }\left(\frac{\lambda x}{2\eta }\right)=0; & \mathop{\mathrm{lim}}\limits_{x\to 0}{F}_{\eta }\left(\frac{\lambda x}{2\eta }\right)ln\,(| \,\lambda \,| x)=0;\quad \frac{\partial {F}_{\eta }\left(\frac{\lambda x}{2\eta }\right)}{| \,\lambda \,| \partial x}=\frac{{C}_{\eta }}{2\eta }\\ \mathrm{the}\,n\,\mathop{\mathrm{lim}}\limits_{x\to 0}{G}_{\eta }\left(\frac{\lambda x}{2\eta }\right)=\frac{1}{{C}_{\eta }} & \mathrm{and}\,\mathop{\mathrm{lim}}\limits_{x\to 0}\frac{\partial {G}_{\eta }\left(\frac{\lambda x}{2\eta }\right)}{| \,\lambda \,| \partial x}+{G}_{\eta }\left(\frac{\lambda x}{2\eta }\right)ln\,(| \,\lambda \,| x)=-\frac{{g}_{f}(\eta )}{{C}_{\eta }};\end{array}$$

so, considering any ${\phi }_{e}(x)={\alpha }_{k}^{\omega }{F}_{\eta }(k\,x)+{\beta }_{\eta }^{\omega }{G}_{\eta }(k\,x)\in {{\mathscr{F}}}_{\omega }$ with ${\beta }_{k}^{\omega }\ne 0$, one gets

$$\begin{array}{lll}\mathop{\mathrm{lim}}\limits_{x\to 0}\frac{{\alpha }_{k}^{\omega }\frac{\partial {F}_{\eta }\left(\frac{\lambda x}{2\eta }\right)}{| \,\lambda \,| \partial x}+{\beta }_{\eta }^{\omega }\frac{\partial {G}_{\eta }\left(\frac{\lambda x}{2\eta }\right)}{| \,\lambda \,| \partial x}}{{\alpha }_{k}^{\omega }{F}_{\eta }\left(\frac{\lambda x}{2\eta }\right)+{\beta }_{\eta }^{\omega }{G}_{\eta }\left(\frac{\lambda x}{2\eta }\right)}+ln\,(| \,\lambda \,| x) & = & {\zeta }_{k}^{\omega }\mathop{\mathrm{lim}}\limits_{x\to 0}\frac{\frac{\partial {F}_{\eta }\left(\frac{\lambda x}{2\eta }\right)}{| \,\lambda \,| \partial x}}{{G}_{\eta }\left(\frac{\lambda x}{2\eta }\right)}+\,\mathop{\mathrm{lim}}\limits_{x\to 0}\frac{\frac{\partial {G}_{\eta }\left(\frac{\lambda x}{2\eta }\right)}{| \,\lambda \,| \partial x}}{{G}_{\eta }\left(\frac{\lambda x}{2\eta }\right)}+ln\,(| \,\lambda \,| x)\\  & = & 2\eta ({g}_{f}(\eta )-{g}_{b}({\eta }_{\omega }))\frac{1}{2\eta }-\,{g}_{f}(\eta )=-{g}_{b}({\eta }_{\omega }).\end{array}$$

while, for ${\beta }_{k}^{\omega }=0$, which corresponds to Rydberg states, one gets

$$\mathop{\mathrm{lim}}\limits_{x\to 0}\frac{\frac{\partial {F}_{\eta }\left(\frac{\lambda x}{2\eta }\right)}{| \,\lambda \,| \partial x}+{F}_{\eta }\left(\frac{\lambda x}{2\eta }\right)ln\,(| \,\lambda \,| x)}{{F}_{\eta }\left(\frac{\lambda x}{2\eta }\right)}=\infty .$$

This proves exactly that *ϕ*_*e*_ belongs to ${\widetilde{{\mathscr{F}}}}_{\omega }$. Reversely, let us show that any element ${\phi }_{e}\in {\widetilde{{\mathscr{F}}}}_{\omega }$ belongs to ${{\mathscr{F}}}_{\omega }$. Using (2), one writes *ϕ*_*e*_ = *α*_*k*_*F*_*η*_ + *β*_*k*_*G*_*η*_. Then, from the definition of ${\widetilde{{\mathscr{F}}}}_{\omega }$, one gets

$${\alpha }_{k}\frac{{C}_{\eta }}{2\eta }-{\beta }_{k}\frac{{g}_{f}(\eta )}{C\eta }={\beta }_{k}\frac{\omega }{{C}_{\eta }}\;\iff \;\left\{\begin{array}{ll}{\mathbb{i}}f{\beta }_{k}\ne 0 & {\zeta }_{k}=\frac{2\eta }{{C}_{\eta }^{2}}\left(\omega +{g}_{f}(\eta )\right);\\ {\mathbb{i}}f{\beta }_{k}=0 & {\zeta }_{k}=\infty ;\end{array}\right.$$

and the scalar product $\left\langle {\varphi }_{{\eta }_{\omega }}| {\phi }_{e}\right\rangle$ reads

$$\left\langle {\varphi }_{{\eta }_{\omega }}| {\phi }_{e}\right\rangle ={\alpha }_{k}\left\langle {\varphi }_{{\eta }_{\omega }}| {F}_{\eta }\right\rangle +{\beta }_{k}\left\langle {\varphi }_{{\eta }_{\omega }}| {G}_{\eta }\right\rangle =\frac{{\mu }_{{k}_{\omega }}{\lambda }^{3/2}}{2({k}_{\omega }^{2}+{k}^{2})\Gamma (1+{\eta }_{\omega })}\left(\frac{{\alpha }_{k}{C}_{\eta }}{2\eta }+\frac{{\beta }_{k}}{{C}_{\eta }}\left(-\omega -{g}_{f}(\eta )\right.\right)=0$$

so ${\phi }_{e}\in {{\mathscr{F}}}_{\omega }$. The case *β*_*k*_ = 0 corresponds to the Rydberg one, *ζ*_*k*_ =∞ = *ω*, and $\left|{\varphi }_{{\eta }_{\infty }}\right\rangle$ is orthogonal to all states in ${{\mathscr{F}}}_{\infty }$. ■

Our classification is coherent with that of ref. ^12^, all self-adjoint extensions of $H({{\mathbb{R}}}_{+}^{\ast })$ are classified by $\omega \in {\mathbb{R}}$. The topology of the parameter space is studied in section ‘Structure for ${\mathbb{D}}={{\mathbb{R}}}_{+}^{\ast }$ in the repulsive case’.

### Classification in the repulsive case

We now consider the repulsive case. The physical situation is very different to the previous one, for instance, one observes that there is no Rydberg state, that is no eigenfunction obeying *ϕ*_*e*_(0) = 0, however many steps of the calculations are similar, so we will only point out the differences.

**Figure 2:**
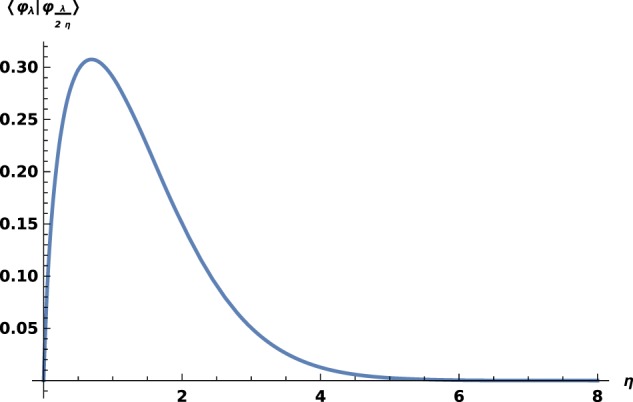
$\langle {\varphi }_{\lambda }| {\varphi }_{\frac{\lambda }{2\eta }}\rangle$ versus *η* in the repulsive case; the choice ${\eta }_{1}=\frac{1}{2}$ is arbitrary, curves obtained for other values are similar.

**Figure 3:**
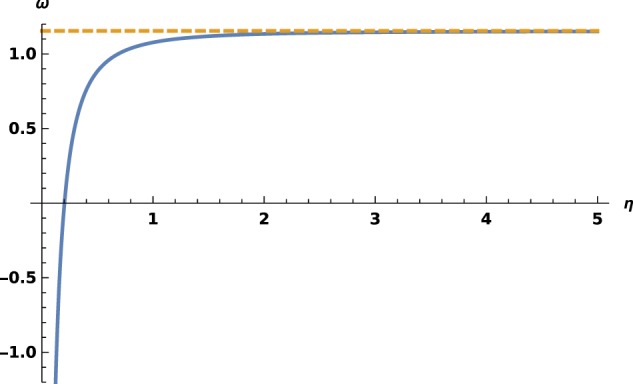
*ω* versus *η* in the repulsive case. The asymptote *ω* = 2*γ*_E_ is drawn with a dashed line.

Keeping the definition of *g*_b_ with *η* > 0, one finds (4) with the opposite sign. Then, (5) has no solutions, but the existence of a bound state will hold in the repulsive case, which means that it is a unique bound state. This is true for all *ω*, see for instance Fig. 2, and confirmed by the bijectivity of *η* ↦ *ω*(*η*), as one observes on Fig. 3. However, (8) extends in the repulsive case, where *η*_*ω*_ stands for the unique bound state in ${H}_{\omega }({{\mathbb{R}}}_{+}^{\ast })$ and the sign is also changed.

In the free spectrum, a similar sign difference occurs:

$$\langle {G}_{{\eta }_{1}}| {G}_{{\eta }_{2}}\rangle =\frac{\lambda }{{C}_{{\eta }_{1}}{C}_{{\eta }_{2}}}\frac{{g}_{f}({\eta }_{1})-{g}_{f}({\eta }_{2})}{{k}_{1}^{2}-{k}_{2}^{2}}+\delta ({k}_{1}-{k}_{2}),$$

where the definition of *g*_f_ is unchanged. The scalar product expression $\left\langle {G}_{{\eta }_{1}}| {F}_{{\eta }_{2}}\right\rangle$ is unchanged but mind that its real sign is also changed after that of *η*. Eventually, the demonstration that all functions in ${{\mathscr{F}}}_{\omega }$ respect $\left\langle {\Phi }_{{e}_{1}}| {\Phi }_{{e}_{2}}\right\rangle$=0 holds, and, consequently, the determination of ${H}_{\omega }({{\mathbb{R}}}_{+}^{\ast })$ is formally identical.

The characterization of ${{\mathscr{F}}}_{\omega }$ is performed with index

$$\omega (\eta )=\mathop{\mathrm{lim}}\limits_{x\to 0}\frac{\frac{\partial {\phi }_{e}\left(\frac{\lambda x}{2\eta }\right)}{\lambda \partial x}-{\phi }_{e}\left(\frac{\lambda x}{2\eta }\right)ln\,(\lambda x)}{{\phi }_{e}\left(\frac{\lambda x}{2\eta }\right)}$$

(note the sign difference). With this new definition, index *ω*(*η*) has the same expression than in the attractive case. The demonstration is straight forward for the bound states; for free ones, one finds

$$\mathop{\mathrm{lim}}\limits_{x\to 0}\frac{\partial {G}_{\eta }\left(\frac{\lambda x}{2\eta }\right)}{\lambda \partial x}-{G}_{\eta }\left(\frac{\lambda x}{2\eta }\right)ln\,(\lambda x)=\frac{{g}_{f}(\eta )}{{C}_{\eta }}\,\mathrm{and}\,\mathop{\mathrm{lim}}\limits_{x\to 0}{G}_{\eta }\left(\frac{\lambda x}{2\eta }\right)=-\frac{1}{{C}_{\eta }};$$

the expression obtained for *F*_*η*_ are unchanged, but mind that the real sign is changed after that of *η*. Eventually, there is no sign change for index *ω*(*η*) in all cases. We plot this function in Fig. 3 and observe another major difference: it maps ${{\mathbb{R}}}_{+}^{\ast }$ on $\left[-\infty ,2{\gamma }_{E}\right]$. As a consequence, *ω* is bounded from above. The particular value *ω* = 2*γ*_E_ brings a very peculiar situation and must be studied elsewhere.

### Existence of a bound state

The classification of self-adjoint extensions of $H({{\mathbb{R}}}_{+}^{\ast })$ is achieved, except that we did not prove the existence of a bound eigenstate $\left|{\varphi }_{{k}_{\omega }}\right\rangle$ of ${H}_{\omega }({{\mathbb{R}}}_{+}^{\ast })$ associated to the eigenvalue *e*_*ω*_ ≤ 0 in both attractive and repulsive cases.

We suppose ad absurdum that the spectrum is included in ${{\mathbb{R}}}_{+}$. We consider two eigenfunctions ${\Psi }_{{k}_{1}}$ and ${\Psi }_{{k}_{2}}$. We can choose momenta *k*_1_ ≠ *k*_2_, otherwise ${H}_{\omega }({{\mathbb{R}}}_{+}^{\ast })$ would only act on functions ${F}_{{\eta }_{1}}$ and ${G}_{{\eta }_{1}}$, which norm are infinite; no integrable function could be constructed and this extension would not be physical. The same argument holds if there is only one eigenfunction.

Using (2) and (9), one gets

$$\left\langle {\Psi }_{{k}_{1}}| {\Psi }_{{k}_{2}}\right\rangle =\frac{\lambda }{{k}_{1}^{2}-{k}_{2}^{2}}\left(-\frac{{C}_{{\eta }_{1}}\bar{{\alpha }_{{k}_{1}}}{\beta }_{{k}_{2}}}{2{\eta }_{1}{C}_{{\eta }_{2}}}+\frac{{C}_{{\eta }_{2}}{\alpha }_{{k}_{2}}\bar{{\beta }_{{k}_{1}}}}{2{\eta }_{2}{C}_{{\eta }_{1}}}+\frac{\bar{{\beta }_{{k}_{1}}}{\beta }_{{k}_{2}}}{{C}_{{\eta }_{1}}{C}_{{\eta }_{2}}}\left({g}_{f}({\eta }_{1})-{g}_{f}({\eta }_{2})\right)\right)=0.$$

If ${\beta }_{{k}_{1}}=0$ and ${\beta }_{{k}_{2}}\ne 0$, one gets ${\alpha }_{{k}_{1}}=0$, which is impossible since ${\Psi }_{{k}_{1}}\ne 0$. So, either both ${\beta }_{{k}_{{\mathbb{i}}}}$ are zero, or both are different from zero. In the first case, this property extends to all free states, which are therefore all Rydberg free ones; thus, ${H}_{\omega }({{\mathbb{R}}}_{+}^{\ast })$ can extend on all standard Rydberg solutions, including bound ones, which contradicts our hypothesis.

The remaining case leads to ${\beta }_{{k}_{{\mathbb{i}}}}\ne 0$ ∀*i* = 1, 2, which means that momenta *k*_*i*_ correspond to non Rydberg states. Multiplying by $\frac{{C}_{{\eta }_{1}}{C}_{{\eta }_{2}}}{\bar{{\beta }_{{k}_{1}}}{\beta }_{{k}_{2}}}$, one gets

10 $$-\frac{{C}_{{\eta }_{1}}^{2}\,\bar{{\zeta }_{{k}_{1}}}}{2{\eta }_{1}}+\frac{{C}_{{\eta }_{2}}^{2}\,{\zeta }_{{k}_{2}}}{2{\eta }_{2}}+{g}_{f}({\eta }_{1})-{g}_{f}({\eta }_{2})=0.$$

One can assume ${\beta }_{{k}_{i}}$ real, without loss of generality. Let us define the real and purely imaginary parts of eigenstates ${\Psi }_{{k}_{i}}$, ${\Psi }_{{k}_{i}}^{r}=\Re ({\Psi }_{{k}_{i}})$ and ${\Psi }_{{k}_{{\mathbb{i}}}}^{i}=\Im ({\Psi }_{{k}_{i}})$. Since (1) is real, both ${\Psi }_{{k}_{i}}^{r}$ and ${\Psi }_{{k}_{i}}^{i}$ are eigenfunctions associated to the same momentum *k*_*i*_. By construction (${\beta }_{{k}_{i}}$ real), ${\Psi }_{{k}_{i}}^{i}\,\propto \,{F}_{{\eta }_{i}}$, which corresponds to a Rydberg state (because ${g}_{b}\left(\frac{\lambda }{2k}\right)=\infty$, cf. section ‘Classification in the attractive case’, in which this item holds both for repulsive or attractive case) and is contradictory, unless ${\Psi }_{{k}_{i}}^{i}=0$. Altogether, this implies that ${\zeta }_{{k}_{i}}$ is real  ∀*i* = 1, 2. Eventually, one gets

11 $$\frac{{C}_{{\eta }_{1}}^{2}\,{\zeta }_{{k}_{1}}}{2{\eta }_{1}}-{g}_{f}({\eta }_{1})=\frac{{C}_{{\eta }_{2}}^{2}\,{\zeta }_{{k}_{2}}}{2{\eta }_{2}}-{g}_{f}({\eta }_{2}),$$

so $\frac{{C}_{\eta }^{2}{\zeta }_{k}}{2\eta }-{g}_{f}(\eta )$ is a real constant, which we write $\widetilde{\omega }$. From the classifications above, one observes that all functions Ψ_*k*_ are eigenfunctions of ${H}_{\widetilde{\omega }}({{\mathbb{R}}}_{+}^{\ast })$, which proves an extension of ${H}_{\omega }({{\mathbb{R}}}_{+}^{\ast })$ and therefore contains bound eigenstates. We have reached a contradiction. In all cases, we have shown that there is at least one bound state. ■

In the repulsive case, it is the only one. In the attractive case, they are infinitely many; let us study that of highest energy.

### Maximum of ${\boldsymbol{\mathscr{S}}}_{{\boldsymbol{\omega }}}$

**Figure 4:**
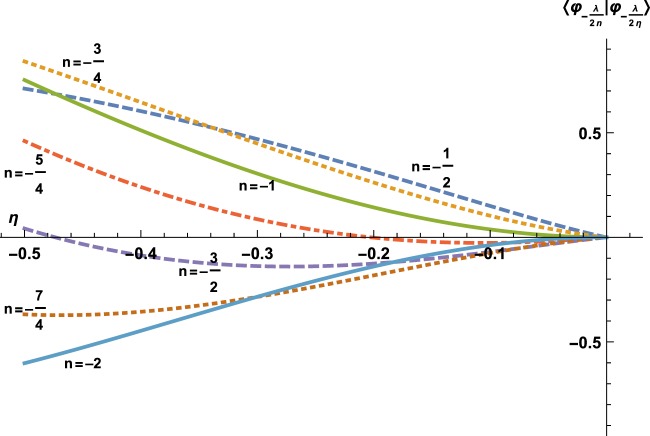
Here is a zoom of Fig. 1 in the interval $\left[-\frac{1}{2},0\right]$.

For the attractive case, *η* < 0, so one is interested in the maximal value *η*_*ω* m*ax*_ corresponding to the maximum of ${{\mathscr{S}}}_{\omega }$. There exists such a maximum, this is visible on Fig. 4, which is a close focus of Fig. 1 in the interval $\left[-\frac{1}{2},0\right]$. To be more precise, the slope of curve $\eta \mapsto \langle {\varphi }_{-\frac{\lambda }{2n}}| {\varphi }_{-\frac{\lambda }{2\eta }}\rangle$ at *η* = 0 reads $\frac{2}{\Gamma (n)}$ which indicates that the curves corresponding to Rydberg eigenstates, $-n\in {{\mathbb{N}}}^{\ast }$, are flat, while the sign of the slope of the other curves is positive for [*n*]_+_ even and negative for [*n*]_+_ odd. Therefore, the maximal *η* < 0, related to an energy $e\in {{\mathscr{S}}}_{\omega }$ is the first zero from the right. The only difficult case would be that of the flat curves; these however correspond to the standard Rydberg solutions $n\in -{{\mathbb{N}}}^{\ast }$, the maximal value of which is indeed  −1. ■

### Infinite energy state

In what precedes we exclude value *η* = 0. Limit *η* → 0 of eigenfunctions corresponding to bound states reads *f*_0_(*u*) = e^−*u*^, but using rescaled *φ*_*k*_(*x*) = *f*_*η*_(*kx*) and renormalizing by *D*_*η*_, one gets

$$\mathop{\mathrm{lim}}\limits_{\eta \to {0}^{\pm }}\left({f}_{\eta }\left(\frac{\lambda x}{2\eta }\right)\right)=0$$

for all $x\in {{\mathbb{R}}}_{+}^{\ast }$ but not for *x* = 0 ( ± = +  in the repulsive case,  ± = − in the attractive one). The so called infinite energy  −∞ would correspond to a singular distribution with {0} support. Looking for such a solution, one substitutes ${\varphi }_{0}={\sum }_{n=0}^{\infty }{a}_{n}{\delta }^{(n)}$ in (1). In the *η* = 0 limit, all coefficients *a*_*n*_ are found zero, which definitely discards such solution.

The limit *η* → 0 of eigenfunctions corresponding to free states reads ${F}_{0}(x)=\sin (x)$ and ${G}_{0}(x)=\cos (x)$. Using rescaled Φ_0_(*x*) = *α*_0_*F*_0_(*kx*) + *β*_0_*G*_0_(*kx*) (but no renormalization is needed, since the limit of *C*_*η*_ is 1), one gets

$$\mathop{\mathrm{lim}}\limits_{\eta \to \infty }{F}_{\pm \eta }\left(\frac{\lambda x}{2\eta }\right)=0\,\mathrm{and}\,\mathop{\mathrm{lim}}\limits_{\eta \to \infty }{G}_{\pm \eta }\left(\frac{\lambda x}{2\eta }\right)=1.$$

The first is zero so the limit of eigenfunctions when *e* → +∞ is the constant function Ψ(*x*) = 1.

Eventually, we should compare these limits to the solutions of (1), where *η* is replaced by 0. They read *ϕ*_∞_(*x*) = *ax* + *b*, but *a* ≠ 0 gives divergent non physical functions, so, up to an arbitrary phase, one finds *b* = 1, which is the *e* = +∞ limit. ■

Incidentally, we are in position to discuss the long-standing claim^1^ of a solution $\left|{\phi }_{-\infty }\right\rangle$ with energy  −∞: we see that this solution does not exist, putting an end to this old story.

### Discussion of some particular cases

#### Dirichlet solutions

We consider the attractive case. When *ω* → ±∞, one gets the Dirichlet condition *ϕ*_*e*_(0) = 0. For bound states, this can be shown by examining the limit *φ*_*k*_(0^+^) = *D*_*η*_/Γ(1 + *η*), which we give in section ‘Classification in the attractive case’ and which is also valid in the repulsive case. For free states, this follows, firstly, from the fact that *ζ*_*k*_ →∞, as shown in the same section, which implies *β*_*k*_ → 0 so *ϕ*_*e*_ ∝ *F*_*η*_, secondly from the limit *F*_*η*_(0^+^) = 0, still proved in that section. Then, the corresponding values of ${{\mathscr{S}}}_{\infty }$ are exactly  −*λ*^2^/(4*n*^2^), for all $n\in {{\mathbb{N}}}^{\ast }$, which is the standard Rydberg spectrum (in dimensionless unit). Moreover, the function *η* ↦ *ω*(*η*) = −*g*_b_(*η*) respects *ω*(*η* + 1) = *ω*(*η*) for all *η* = −*n* with $n\in {{\mathbb{N}}}^{\ast }$ and only for these values.

In the repulsive case, one must recall that there is no Rydberg state, even in the limit *ω* → −∞, so this discussion is not relevant for this case.

#### Neumann solutions

The case *ω* = 0 will be called the Neumann solutions, because the finite part^18^ of ${\phi }_{e}^{{\prime} }\in {{\mathscr{D}}}_{0}$, where the essential divergent function $ln\,(k\,x)$ is left aside, is exactly zero at *x* = 0. These functions are very close to the anomalous solutions of ref. ^11^, however those do not belong to a single extension: they are proportional to *G*_*η*_ in the free spectrum and correspond to *ζ*_*k*_ = 0. We have shown previously that ${\zeta }_{k}=\frac{2\eta }{{C}_{\eta }^{2}}({g}_{f}(\eta )-{g}_{b}(\eta ))$, which zeros are not exactly periodic, on the contrary, each one belongs to a different extension. The very small difference between any such anomalous state and the closest Neumann one explains the small violation of orthogonality that was calculated^11^ (when *η* →∞, the difference between Neumann and anomalous solutions tends to zero, as well as the scalar products between anomalous solutions).

As is well understood now, the correct choice is to consider functions in ${{\mathscr{B}}}_{0}$. On the contrary, it is not physical to consider any two anomalous states together^19^, because they do not belong to the same self-adjoint extension.

#### Physical interpretation of *ω*

We did not give any physical interpretation of *ω* yet. It is the limit of the ratio $\frac{\partial \phi (x)}{| \,\lambda \,| \partial x}/\phi (x)$ between the derivative of the wavefunction and the wavefunction itself when *x* → 0, **after subtracting the divergent term** $\pm ln\,(| \,\lambda \,| x)$ ( ± = +  when the potential is attractive,  ± = − when it is repulsive).

This ratio relates to the initial condition that one fixes at *x* = 0 when solving Schrödinger equation *Hϕ* = *Eϕ*. An infinite ratio corresponds to choosing Dirichlet conditions, a zero ratio to Neumann ones, and any finite value in-between means fixing an intermediate condition, that mixes *ϕ* and $\phi {\prime}$.

#### Solutions of zero energy

Writing ${{\mathbb{R}}}_{+}$, we have indicated that 0 must be included in the free spectrum. This is worth giving some details.

The solutions of (1) for *e* = 0 and *λ* < 0 read

$${\Psi }_{0}(x)=\alpha j(x)+\beta y(x);\quad j(x)=\sqrt{| \,\lambda \,| x}{J}_{1}\left(2\sqrt{| \,\lambda \,| x}\right);\quad y(x)=\sqrt{| \,\lambda \,| x}{Y}_{1}\left(2\sqrt{| \,\lambda \,| x}\right),$$

where *J*_1_ and *Y*_1_ are Bessel functions of, respectively, the first and second kind. That for *λ* > 0 read

$${\Psi }_{0}(x)=\alpha \,\iota (x)+\beta \,\kappa (x);\quad \iota (x)=\sqrt{| \,\lambda \,| x}{i}_{1}\left(2\sqrt{| \,\lambda \,| x}\right);\quad \kappa (x)=\sqrt{| \,\lambda \,| x}{K}_{1}\left(2\sqrt{| \,\lambda \,| x}\right),$$

where *I*_1_ and *K*_1_ are modified Bessel functions of, respectively, the first and second kind.

We have extended the notations we use for free states, because these solutions are indeed the limit of those ones, *j* ∝ *F*_−∞_, *y* ∝ *G*_−∞_, *ι* ∝ *F*_∞_ and *κ* ∝ *G*_∞_. The attractive case *η* < 0 brings nothing special, solutions *j* and *y* have the standard properties of the eigenfunctions corresponding to free states; one may say that this limit is regular.

On the contrary, the repulsive case *η* > 0 is extraordinary. Instead of heavy mathematical considerations, let us explain the situation by hand. When one looks at the curves of functions *x* ↦ *F*_*η*_(*x*) and *x* ↦ *G*_*η*_(*x*), for increasing *η*, one observes that there are two regions *x* ∈ [0, *x*_*η*_] and $x\in \left[{x}_{\eta },\infty \right.\left[\right.$, where *x*_*η*_ is a separating parameter which we do not care to define properly here. In region [0, *x*_*η*_], *F*_*η*_ resembles eigenfunction *g*_*η*_ (in other words, it grows considerably, as if it were diverging) and *G*_*η*_ resembles eigenfunction *f*_*η*_ (in other words, it becomes exponentially small). But, as these functions reach *x*_*η*_, they rapidly change shape and behave like those corresponding to standard free states (bounded and oscillating).

**Figure 5:**
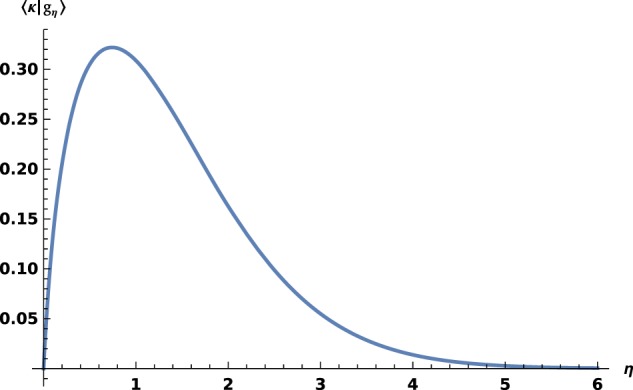
〈*κ*∣*g*_*η*_〉 versus *η*.

This peculiar behavior, resembling bound states in a first region then free ones afterwards, reaches its climax when *η* →∞, where *x*_*η*_ →∞: indeed, solution *ι* is diverging, while $\kappa \in {L}^{1}({{\mathbb{R}}}_{+}^{\ast })\bigcap {L}^{2}({{\mathbb{R}}}_{+}^{\ast })$. In this very case, *F*_∞_ must be discarded and the scalar products between *G*_∞_ and eigenfunctions *f*_*η*_ reads

$$\left\langle \kappa | {f}_{\eta }\right\rangle =\frac{2\eta {D}_{\eta }}{\Gamma (1+\eta )}\left(1+2\eta \left(ln\,(\eta )-\Gamma (1+\eta )\right)\right)$$

and is non zero, as observed on Fig. 5. The orthogonal combination of eigenfunctions *F*_*η*_ and *G*_*η*_ is governed by ratio

$$\frac{{\alpha }_{\eta }^{\infty }}{{\beta }_{\eta }^{\infty }}=\frac{2\eta }{{C}_{\eta }^{2}}\left(\Re \left(\Gamma (1-{\mathbb{i}}\eta )\right)-ln\,(\eta )\right).$$

Our guess is that, in the repulsive case, a singular contribution *δ*(*E*) appears in the density of states, contrary to the situation of the attractive case. This belief is founded by the existence of a bound eigenstate, to which corresponds an integrable function, with eigenvalue *e* = 0.

Eventually, one is interested in the corresponding value of index *ω*(∞ ). One finds *ω*(∞ ) = 2*γ*_E_. Moreover, the limit of regular bound eigenfunction *φ*_*k*_, when *η* →∞, does not exist, so there is exactly one bound eigenstate of energy *e* = 0 corresponding to *ω*(∞ ) = 2*γ*_E_, which is exactly that proportional to *κ*.

## The real line problem

We discuss here the attractive case for ${\mathbb{D}}={\mathbb{R}}$. We should point out that there was no need to use of any physical constraint in the previous cases, except when we have discarded the hypothesis of a unique energy *e* > 0 or that with only two energies *e*_1_ > *e*_2_ > 0. On the contrary, our determination of self-adjoint extensions for ${\mathbb{D}}={\mathbb{R}}$ is much more involved with physical laws. Our aim is to classify self-adjoint extensions that are compatible with physical constraints.

We note *ϕ*_*e*_ eigenfunctions defined on ${\mathbb{D}}$, ${\phi }_{e}^{ > }$ their restriction on ${{\mathbb{R}}}_{+}^{\ast }$ and ${\phi }_{e}^{ < }$ that on ${{\mathbb{R}}}_{-}^{\ast }$. ${\phi }_{e}^{ > }$ obeys (1), while ${\phi }_{e}^{ < }$ obeys

12 $$-\frac{{\partial }^{2}{\phi }_{e}}{\partial {x}^{2}}(x)-\frac{\lambda }{x}{\phi }_{e}(x)=e{\phi }_{e}(x)\quad \forall x < 0.$$

The continuity of all functions *ϕ*_*e*_ as well as their derivatives is easily verified for all *x* ≠ 0 from (1) and (12). The only difficulty lies at *x* = 0. Let us define the self-adjoint extensions of $H({\mathbb{R}})$.

### Self-adjoint extensions

The mathematical classification of all self-adjoint extensions, for ${\mathbb{D}}={\mathbb{R}}$, has already been done^12^ but no effort has been made yet to interpret these from a physical point of view. We want to select, among all extensions, only those, the eigenfunctions of which describe physical states.

Usually, authors impose continuous boundary conditions for all wavefunctions and their derivative^20–23^ but these conditions reveal often too restrictive and other boundary conditions have been suggested^24,25^. So, we choose weaker and universal constraints, which are compatible with any of these conditions and fit with all experimental observations: the density of probability cannot vary discontinuously, therefore *ρ* = ∣*ϕ*∣^2^ must be continuous. *ρ* also obeys the conservation of probability law (14). This implies eventually that *d***j**/*dx* be defined at all $x\in {\mathbb{R}}$.

We introduce boundary condition ${\mathscr{C}}(\theta )$:

$$\left.\begin{array}{c}\mathop{\mathrm{lim}}\limits_{\varepsilon \to 0}\left(\phi (\varepsilon )-{{\rm{e}}}^{{\mathbb{i}}\theta }\phi (-\varepsilon )\right)=0;\\ \mathop{\mathrm{lim}}\limits_{\varepsilon \to 0}\left(\phi {\prime} (\varepsilon )\phi (-\varepsilon )-{{\rm{e}}}^{{\mathbb{i}}\theta }\phi {\prime} (-\varepsilon )\phi (\varepsilon )\right)=0;\end{array}\right\}\quad \theta \in \left[0,2\pi \right.\left[\right.;$$

we will find that physical states do respect conditions ${\mathscr{C}}(\theta )$. We will therefore construct self-adjoint extensions, with these boundary conditions. More precisely, we will show that there are at maximum two values *θ*_1_ and *θ*_2_, such that eigenfunctions obey ${\mathscr{C}}({\theta }_{i})$, with *i* = 1, 2.

As for $D={{\mathbb{R}}}_{+}^{\ast }$, we will admit the existence of self-adjoint extensions and construct them as maximal symmetric operators. We write them ${H}_{\varpi }({\mathbb{R}})$, where *ϖ* is a symbolic parameter, the meaning of which we will clarify further on. We write ${{\mathscr{B}}}_{\varpi }$ the set of eigenfunctions in the bound spectrum, ${{\mathscr{F}}}_{\varpi }$ that of eigenfunctions in the free spectrum, ${{\mathscr{D}}}_{\varpi }={{\mathscr{B}}}_{\varpi }\bigcup {{\mathscr{F}}}_{\varpi }$ and ${{\mathscr{S}}}_{\varpi }$ the corresponding bound spectrum.

### Continuity of probability

Let *ϕ*_*e*_ be an eigenfunction of self-adjoint extension ${H}_{\varpi }({\mathbb{R}})$. We will first use the continuity of *ρ*(*x*) = ∣*ϕ*_*e*_(*x*)∣^2^.

One put apart the case when *ϕ*_*e*_(0^+^) = 0 or *ϕ*_*e*_(0^−^) = 0. Indeed, the only eigenfunctions which have such limit are the Rydberg ones. In such case, the continuity of *ρ* gives *ϕ*_*e*_(0^+^) = *ϕ*_*e*_(0^−^) = 0 and *ϕ*_*e*_ is eventually continuous on ${\mathbb{R}}$.

We recall that *non Rydberg* functions do not cancel at *x* = 0. For such functions, the continuity of *ρ* implies ∣*ϕ*_*e*_(0^−^)∣ = ∣*ϕ*_*e*_(0^+^)∣ ⇔ *ϕ*_*e*_(0^−^) = e^i*θ*^*ϕ*_*e*_(0^+^) with $\theta \in \left[0,2\pi \right.\left[\right.$.

Let ${\phi }_{{e}_{1}}$ and ${\phi }_{{e}_{2}}\in {{\mathscr{D}}}_{\varpi }$ be two independent eigenfunctions, ${\phi }_{{e}_{1}}({0}^{-})={{\rm{e}}}^{i{\theta }_{1}}{\phi }_{{e}_{1}}({0}^{+})$ and ${\phi }_{{e}_{2}}({0}^{-})={{\rm{e}}}^{i{\theta }_{2}}{\phi }_{{e}_{2}}({0}^{+})$. $\left|{\phi }_{{e}_{1}}\right\rangle$ and $\left|{\phi }_{{e}_{2}}\right\rangle$ are eigenstates of hermitian operator ${H}_{\varpi }({\mathbb{R}})$, their combination is physical; one can consider state $\left|\psi \right\rangle =\alpha \left|{\phi }_{{e}_{1}}\right\rangle +\beta {{\rm{e}}}^{i\zeta }\left|{\phi }_{{e}_{2}}\right\rangle$ with arbitrary coefficients $(\alpha ,\beta )\in {{\mathbb{R}}}^{2}$ and $\xi \in \left[0,2\pi \right.\left[\right.$. The evolution in time of $\left|\psi \right\rangle$ is given by

$$\left|\psi (t)\right\rangle =\alpha {{\rm{e}}}^{-{\mathbb{i}}\frac{{e}_{1}\hslash t}{2m}}\left|{\phi }_{{e}_{1}}\right\rangle +\beta {{\rm{e}}}^{{\mathbb{i}}\zeta }{{\rm{e}}}^{-{\mathbb{i}}\frac{{e}_{2}\hslash t}{2m}}\left|{\phi }_{{e}_{2}}\right\rangle .$$

*ρ*(*x*, *t*) = ∣*ψ*(*x*, *t*)∣^2^ represents a density of probability and must be continuous with respect to *x* at all times. One finds

$$\begin{array}{lll}\rho (x,t) & = & | \alpha {| }^{2}| {\phi }_{{e}_{1}}(x){| }^{2}+| \beta {| }^{2}| {\phi }_{{e}_{2}}(x){| }^{2}\\  &  & +\,2\alpha \beta \,\Re \left[\bar{{\phi }_{{e}_{1}}(x)}{\phi }_{{e}_{2}}(x)\right]\cos \left[\frac{({e}_{1}-{e}_{2})\hslash t}{2m}+\zeta \right]-\,2\alpha \beta \,\Im \left[\bar{{\phi }_{{e}_{1}}(x)}{\phi }_{{e}_{2}}(x)\right]\sin \left[\frac{({e}_{1}-{e}_{2})\hslash t}{2m}+\zeta \right].\end{array}$$

The continuity of *x* ↦ *ρ*(*x*, *t*), valid for all *α*, *β*, *ζ* and *t*, implies that of $\Re \left(\bar{{\phi }_{{e}_{1}}}{\phi }_{{e}_{2}}\right)$ and $\Im \left(\bar{{\phi }_{{e}_{1}}}{\phi }_{{e}_{2}}\right)$; so one gets

$$\bar{{\phi }_{{e}_{1}}({0}^{+})}{\phi }_{{e}_{2}}({0}^{+})=\bar{{\phi }_{{e}_{1}}({0}^{-})}{\phi }_{{e}_{2}}({0}^{-}).$$

If one of $\{{\phi }_{{e}_{1}},{\phi }_{{e}_{2}}\}$ is *Rydberg* and cancels at *x* = 0, this relation is always true. If they are both non Rydberg, it reads ${{\rm{e}}}^{i({\theta }_{1}-{\theta }_{2})}=1\;\iff \;{\theta }_{1}={\theta }_{2}(2\pi )$, where (2*π*) means modulo 2*π*.

Eventually, we have proved the existence of ${\theta }_{\varpi }\in \left[0,2\pi \right]$ such that, for all non Rydberg eigenfunctions,

13 $${\phi }_{e}({0}^{-})={{\rm{e}}}^{{\mathbb{i}}{\theta }_{\varpi }}{\phi }_{e}({0}^{+}).\quad $$

### *θ*-symmetry

We still consider ${H}_{\varpi }({\mathbb{R}})$. We still assume there exists a non Rydberg eigenfunction *ϕ*_*e*_ in the bound spectrum (*e* < 0). From (3), one can write ${\phi }_{e}^{ > }={\mu }_{k}^{+}{f}_{\eta }$ and ${\phi }_{e}^{ < }={\mu }_{k}^{-}\widehat{{f}_{\eta }}$, where the transposition is defined by $\widehat{\varphi }(x)=\varphi (-x)$. Then, (13) implies ${\mu }_{k}^{-}={e}^{i{\theta }_{\varpi }}{\mu }_{k}^{+}$. Thus, *ϕ*_*e*_ is said to be *θ*_*ϖ*_-symmetrical, where *θ*-symmetry is also written ${\mathcal{R}}(\theta )$ and defined by

$${\mathcal{R}}(\theta ):\qquad {\phi }_{e}={\phi }_{e}^{ > }+{{\rm{e}}}^{i\theta }\widehat{{\phi }_{e}^{ > }}.$$

We assume now that there are two or more non Rydberg eigenfunctions in the bound spectrum, let us write them ${\varphi }_{{k}_{1}}$ and ${\varphi }_{{k}_{2}}$. Note that ${\varphi }_{{k}_{1}}\,\propto \,{\varphi }_{k2}\;\iff \;{\varphi }_{{k}_{1}}^{ > }\,\propto \,{\varphi }_{k2}^{ > }$ (where ${\varphi }_{k}^{ > }$ is the restriction on ${{\mathbb{R}}}_{+}^{\ast }$). Their scalar product reads

$$\langle {\varphi }_{{k}_{1}}| {\varphi }_{{k}_{2}}\rangle =2\langle {\varphi }_{{k}_{1}}^{ > }| {\varphi }_{{k}_{2}}^{ > }\rangle .$$

When they are not proportional, ${\varphi }_{{k}_{1}}$ and ${\varphi }_{{k}_{2}}$ can be eigenfunctions of the same ${H}_{\varpi }({\mathbb{R}})$ only if ${\varphi }_{{k}_{1}}^{ > }$ and ${\varphi }_{{k}_{2}}^{ > }$, their restriction on ${{\mathbb{R}}}_{+}^{\ast }$, are orthogonal each other. From part ‘Self-adjoint extensions in the ${{\mathbb{R}}}_{+}^{\ast }$ case’, we get *ω*(*η*_1_) = *ω*(*η*_2_). Let us call *ω*_*ϖ*_ this constant. Altogether, we have established the existence of parameters *ω*_*ϖ*_ and *θ*_*ϖ*_, such that all non Rydberg eigenfunctions *ϕ*_*e*_, in the bound spectrum, obey ${\mathcal{R}}({\theta }_{\varpi })$ and *g*_b_(*η*) = −*ω*_*ϖ*_, with $\eta =\lambda /(2\sqrt{-e})$, so ${\phi }_{e}^{ > }={\varphi }_{k}^{ > }\in {{\mathscr{B}}}_{{\omega }_{\varpi }}$. ■

We will examine now the situation, where there is also a Rydberg eigenstate in the domain of ${H}_{\varpi }({\mathbb{R}})$, and prove that this Rydberg states has the opposite symmetry to the non Rydberg one, in the following sense. Consider ${\phi }_{{e}_{1}}\in {{\mathscr{B}}}_{\varpi }$, with ${\phi }_{{e}_{1}}(0)\ne 0$, and ${\phi }_{{e}_{2}}\in {{\mathscr{D}}}_{\varpi }$, with ${\phi }_{{e}_{2}}(0)=0$. ${\phi }_{{e}_{1}}$ obeys ${\mathcal{R}}({\theta }_{\varpi })$, which reads ${\phi }_{{e}_{1}}^{\, < \,}={{\rm{e}}}^{i{\theta }_{\varpi }}{\phi }_{{e}_{1}}^{ > }$. One can expand ${\phi }_{{e}_{2}}$ into a *θ*_*ϖ*_-symmetrical and a *θ*_*ϖ*_ + *π*-symmetrical parts, ${\phi }_{{e}_{2}}={\phi }_{e2}^{{\theta }_{\varpi }}+{\phi }_{e2}^{{\theta }_{\varpi }+\pi }$, as demonstrated in Appendix (see Supplementary Information). Then, one finds ${\phi }_{e2}^{{\theta }_{\varpi }}=0$, writing

$$0=\langle {\phi }_{{e}_{1}}| {\phi }_{{e}_{2}}\rangle =\langle {\phi }_{{e}_{1}}| {\phi }_{e2}^{{\theta }_{\varpi }}\rangle +\mathop{\underbrace{\left\langle {\phi }_{{e}_{1}}| {\phi }_{e2}^{{\theta }_{\varpi }+\pi }\right\rangle }}\limits_{\begin{array}{c}=0\end{array}}=2\langle {\phi }_{{e}_{1}}^{ > }| {\phi }_{e2}^{{\theta }_{\varpi } > }\rangle $$

(the second term is zero by symmetry, cf. appendix) so ${\phi }_{e2}^{{\theta }_{\varpi } > }$ is orthogonal to ${\phi }_{{e}_{1}}^{ > }$, which is impossible, since ${\phi }_{{e}_{1}}^{ > }\in {{\mathscr{D}}}_{{\omega }_{\varpi }}$ and ${\phi }_{e2}^{{\theta }_{\varpi } > }\in {{\mathscr{D}}}_{\infty }$ because it is a Rydberg eigenfunction, unless ${\phi }_{e2}^{{\theta }_{\varpi } > }=0$. This proves that ${\phi }_{{e}_{2}}^{{\theta }_{\varpi }}=0$ so ${\phi }_{{e}_{2}}$ obeys ${\mathcal{R}}({\theta }_{\varpi }+\pi )$. ■

Let us examine now free states. We consider a non Rydberg eigenfunction *ϕ*_*e*_ with *e* > 0. We will find that *ϕ*_*e*_ obeys ${\mathcal{R}}({\theta }_{\varpi })$ and that ${\phi }_{e}^{ > }={\Psi }_{k}^{ > }\in {{\mathscr{F}}}_{{\omega }_{\varpi }}$, but the demonstration is more involved and relies also on the current continuity. To begin with, following (2), one can write ${\phi }_{e}^{ > }={\alpha }_{k}^{+}{F}_{\eta }+{\beta }_{k}^{+}{G}_{\eta }$ and ${\phi }_{e}^{ < }={\alpha }_{k}^{-}\widehat{{F}_{\eta }}+{\beta }_{k}^{-}\widehat{{G}_{\eta }}$. Applying (13), one gets ${\beta }_{k}^{-}={{\rm{e}}}^{i{\theta }_{\varpi }}{\beta }_{k}^{+}$.

### Conservation of current

We still consider ${H}_{\varpi }({\mathbb{R}})$ and two independent eigenfunctions ${\phi }_{{e}_{1}}$ and ${\phi }_{{e}_{2}}$ in the domain of ${H}_{\varpi }({\mathbb{R}})$ and calculate the current associated to the mixed state $\left|\psi (t)\right\rangle$ defined in section ‘Continuity of probability’. It becomes, after some calculation,

$$\begin{array}{rcl}{\bf{j}} & = & {{\bf{j}}}_{1}+{{\bf{j}}}_{2}+\frac{\hslash \alpha \beta }{m}\Re \left[\bar{{\phi }_{{e}_{1}}(x)}\frac{\partial {\phi }_{{e}_{2}}}{\partial x}(x)-{\phi }_{{e}_{2}}(x)\bar{\frac{\partial {\phi }_{{e}_{1}}}{\partial x}(x)}\right]\times \sin \left[\frac{({e}_{1}-{e}_{2})\hslash t}{2m}+\zeta \right]\\  &  & +\frac{\hslash \alpha \beta }{m}\Im \left[\bar{{\phi }_{{e}_{1}}(x)}\frac{\partial {\phi }_{{e}_{2}}}{\partial x}(x)-{\phi }_{{e}_{2}}(x)\bar{\frac{\partial {\phi }_{{e}_{1}}}{\partial x}(x)}\right]\times \cos \left[\frac{({e}_{1}-{e}_{2})\hslash t}{2m}+\zeta \right],\end{array}$$

where **j**_1_ and **j**_2_ are constant. The conservation of probability law

14 $$\frac{\partial {\bf{j}}}{\partial x}+\frac{\partial \rho }{\partial t}=0$$

applies independently on the sinus and cosine terms, so it eventually reads

$$\bar{{\phi }_{{e}_{1}}(x)}\frac{{\partial }^{2}{\phi }_{{e}_{2}}}{\partial {x}^{2}}(x)-{\phi }_{{e}_{2}}(x)\bar{\frac{{\partial }^{2}{\phi }_{{e}_{1}}}{\partial {x}^{2}}(x)}+({e}_{2}-{e}_{1})\,\bar{{\phi }_{{e}_{1}}(x)}{\phi }_{{e}_{2}}(x)=0$$

and must be verified $\forall x\in {\mathbb{R}}$. For $x\in {{\mathbb{R}}}_{+}^{\ast }$, (14) ⇔ (1); for $x\in {{\mathbb{R}}}_{-}^{\ast }$, (14) ⇔ (12); so, a particular attention must be paid to the determination of ∂**j**/∂*x* when it is evaluated through *x* = 0. One has

$$\frac{\partial {\bf{j}}}{\partial x}(0)=\mathop{\mathrm{lim}}\limits_{\genfrac{}{}{0.0pt}{}{{\varepsilon }_{1}\to {0}^{+}}{{\varepsilon }_{2}\to {0}^{+}}}\frac{{\bf{j}}({\varepsilon }_{2})-{\bf{j}}(-{\varepsilon }_{1})}{{\varepsilon }_{2}+{\varepsilon }_{1}}.$$

Let us continue the proof concerning non Rydberg free states, which was sketched in the previous section. We choose $\left|{\phi }_{{e}_{1}}\right\rangle$ a non Rydberg bound state and $\left|{\phi }_{{e}_{2}}\right\rangle$ a non Rydberg free one (we assume their existence; one observes that they are independent). So ${\phi }_{{e}_{1}}^{ > }={\mu }_{{k}_{1}}^{+}{f}_{{\eta }_{1}}$, ${\phi }_{{e}_{1}}^{\, < \,}={\mu }_{{k}_{1}}^{-}\widehat{{f}_{{\eta }_{1}}}$, ${\phi }_{{e}_{2}}^{ > }={\alpha }_{{k}_{2}}^{+}{F}_{{\eta }_{2}}+{\beta }_{{k}_{2}}^{+}{G}_{{\eta }_{2}}$ and ${\phi }_{{e}_{2}}^{\, < \,}={\alpha }_{{k}_{2}}^{-}\widehat{{F}_{{\eta }_{2}}}+{\beta }_{{k}_{2}}^{-}\widehat{{G}_{{\eta }_{2}}}$, with ${\mu }_{{k}_{1}}^{-}={{\rm{e}}}^{i{\theta }_{\varpi }}{\mu }_{{k}_{1}}^{+}$ and ${\beta }_{{k}_{2}}^{-}={{\rm{e}}}^{i{\theta }_{\varpi }}{\beta }_{{k}_{2}}^{+}$. All terms in the previous limit read $\frac{\hslash \alpha \beta }{m}\Re (\bar{\alpha }\beta ..)\sin (..)+\left(\Re \leftrightarrow \Im \,\& \,\sin \leftrightarrow \cos \right)$. One applies again the independence of sinus and cosine, and skips factor $\frac{\hslash \alpha \beta }{m}$. The first order of the remaining term reads

$$\frac{{D}_{{\eta }_{1}}{C}_{{\eta }_{2}}}{2{\eta }_{2}\Gamma (1+{\eta }_{1})}\mathop{\mathrm{lim}}\limits_{\genfrac{}{}{0.0pt}{}{{\varepsilon }_{1}\to {0}^{+}}{{\varepsilon }_{2}\to {0}^{+}}}\frac{\bar{{\mu }_{{k}_{1}}^{+}}{\alpha }_{{k}_{2}}^{+}-\bar{{\mu }_{{k}_{1}}^{-}}{\alpha }_{{k}_{2}}^{-}}{{\varepsilon }_{1}+{\varepsilon }_{2}}$$

and exists if and only if $\bar{{\mu }_{{k}_{1}}^{+}}{\alpha }_{{k}_{2}}^{+}=\bar{{\mu }_{{k}_{1}}^{-}}{\alpha }_{{k}_{2}}^{-}$ which therefore gives ${\alpha }_{{k}_{2}}^{-}={{\rm{e}}}^{{\mathbb{i}}{\theta }_{\varpi }}{\alpha }_{{k}_{2}}^{+}$. The second order reads

$$-\frac{{D}_{{\eta }_{1}}({\eta }_{1}^{2}+{\eta }_{2}^{2})}{4{\eta }_{1}^{2}{\eta }_{2}^{2}{C}_{{\eta }_{2}}\Gamma (1+{\eta }_{1})}\mathop{\mathrm{lim}}\limits_{\genfrac{}{}{0.0pt}{}{{\varepsilon }_{1}\to {0}^{+}}{{\varepsilon }_{2}\to {0}^{+}}}\frac{\bar{{\mu }_{{k}_{1}}^{+}}{\beta }_{{k}_{2}}^{+}{\varepsilon }_{2}+\bar{{\mu }_{{k}_{1}}^{-}}{\beta }_{{k}_{2}}^{-}{\varepsilon }_{1}}{{\varepsilon }_{1}+{\varepsilon }_{2}}$$

and exists if and only if $\bar{{\mu }_{{k}_{1}}^{+}}{\beta }_{{k}_{2}}^{+}=\bar{{\mu }_{{k}_{1}}^{-}}{\beta }_{{k}_{2}}^{-}$ which therefore gives ${\beta }_{{k}_{2}}^{-}={{\rm{e}}}^{{\mathbb{i}}{\theta }_{\varpi }}{\beta }_{{k}_{2}}^{+}$. We have proved that all non Rydberg obey ${\mathcal{R}}({\theta }_{\varpi })$, although we have not determined the set to which belongs ${\phi }_{e}^{ > }$ when *e* > 0. ■

Before taking advantage of this result, let us conclude on the current of probability. For ${\phi }_{{e}_{1}}$ and ${\phi }_{{e}_{2}}$ non Rydberg, **j** is odd and the limit of **j**(*x*)/*x* when *x* → 0 becomes

$$\begin{array}{rcl}\frac{d{\bf{j}}}{dx}(0) & = & \frac{\bar{{\beta }_{{k}_{1}}^{+}}{\beta }_{{k}_{2}}^{+}}{{C}_{{\eta }_{1}}{C}_{{\eta }_{2}}}\left(\frac{1}{{(2{\eta }_{2})}^{2}}-\frac{1}{{(2{\eta }_{1})}^{2}}\right)\quad \mathrm{if}\,{e}_{1} > 0\,\mathrm{and}\,{e}_{2} > 0;\\  & = & \frac{\bar{{\mu }_{{k}_{1}}^{+}}{\beta }_{{k}_{2}}^{+}{D}_{{\eta }_{2}}}{{C}_{{\eta }_{1}}\Gamma (1+{\eta }_{2})}\left(\frac{1}{{(2{\eta }_{2})}^{2}}+\frac{1}{{(2{\eta }_{1})}^{2}}\right)\quad \mathrm{if}\,{e}_{1} > 0\,\mathrm{and}\,{e}_{2} < 0;\\  & = & \frac{\bar{{\mu }_{{k}_{1}}^{+}}{\mu }_{{k}_{2}}^{+}{D}_{{\eta }_{1}}{D}_{{\eta }_{2}}}{\Gamma (1+{\eta }_{1})\Gamma (1+{\eta }_{2})}\left(\frac{1}{{(2{\eta }_{2})}^{2}}-\frac{1}{{(2{\eta }_{1})}^{2}}\right)\quad \mathrm{if}\,{e}_{1} < 0\,\mathrm{and}\,{e}_{2} < 0.\end{array}$$

This calculation is valid in both attractive or repulsive cases. For Rydberg states, the same three limits give zero (the case *e*_1_ < 0 and *e*_2_ < 0 extends exactly; the case *e*_1_ > 0 and *e*_2_ < 0 also extends, because the wrong normalisation vanishes in the zero limit; the case *e*_1_ > 0 and *e*_2_ > 0 is apart). Altogether, (14) is respected at all cases. ■

### Self-adjoint extensions

We still consider self-adjoint extension ${H}_{\varpi }({\mathbb{R}})$. We assume first that there exists a non Rydberg bound eigenfunction ${\phi }_{{e}_{1}}$. We have shown that there are two parameters *ω*_*ϖ*_ and *θ*_*ϖ*_ such that it reads ${\phi }_{{e}_{1}}^{ > }={\varphi }_{{k}_{1}}^{ > }$ and ${\phi }_{{e}_{1}}^{\, < \,}={{\rm{e}}}^{i{\theta }_{\varpi }}\widehat{{\varphi }_{{k}_{1}}^{ > }}$ with ${g}_{b}\left(\frac{\lambda }{2{k}_{1}}\right)=-{\omega }_{\varpi }$ and ${\varphi }_{{k}_{1}}^{ > }\in {{\mathscr{B}}}_{{\omega }_{\varpi }}$. In other words, ${\phi }_{{e}_{1}}$ is a *θ*-symmetrical eigenfunction of ${{\mathscr{B}}}_{\varpi }$.

Let us achieve the proof concerning non Rydberg free states; so we assume there is such an eigenfunction ${\phi }_{{e}_{2}}$, with *e*_2_ > 0. We know ${\phi }_{{e}_{2}}$ obeys ${\mathcal{R}}({\theta }_{\varpi })$. So the scalar product $\langle {\phi }_{{e}_{1}}| {\phi }_{{e}_{2}}\rangle$ reads

$$0=\langle {\varphi }_{{e}_{1}}| {\phi }_{{e}_{2}}\rangle =2\langle {\phi }_{{e}_{1}}^{ > }| {\phi }_{e2}^{ > }\rangle ;$$

it is zero because they are both eigenfunctions of the same operator ${H}_{\varpi }({\mathbb{R}})$. Now, the equality ${\widetilde{{\mathscr{F}}}}_{{\omega }_{\varpi }}={{\mathscr{F}}}_{{\omega }_{\varpi }}$ implies $\left\{\left|{\phi }_{{e}_{1}}^{ > }\right\rangle \perp \left|{\phi }_{{e}_{2}}^{ > }\right\rangle \;\iff \;{\phi }_{{e}_{2}}^{ > }\in {{\mathscr{F}}}_{{\omega }_{\varpi }}\right\}$. This proves that ${\phi }_{{e}_{2}}$ obeys ${\mathcal{R}}({\theta }_{\varpi })$ with ${\phi }_{{e}_{2}}^{ > }\in {{\mathscr{F}}}_{{\omega }_{\varpi }}$. Altogether, all non Rydberg states obey ${\mathcal{R}}({\theta }_{\varpi })$ with ${\phi }_{e}^{ > }\in {{\mathscr{D}}}_{{\omega }_{\varpi }}$. ■

Let us eventually consider any Rydberg eigenfunction ${\phi }_{{e}_{3}}$ of the same operator ${H}_{\varpi }({\mathbb{R}})$. We know that this function is *θ*_*ϖ*_ + *π*-symmetrical. Reversely, all *θ*_*ϖ*_ + *π*-symmetrical Rydberg eigenfunctions are orthogonal to any (here non Rydberg) *θ*_*ϖ*_-symmetrical function (cf. Appendix in Supplementary Information), so ${H}_{\varpi }({\mathbb{R}})$ can be extended into a symmetric operator (one can choose a trivial action ${H}_{\varpi }({\mathbb{R}})\left|{\phi }_{e}\right\rangle =\left|0\right\rangle$), acting on all eigenstates *ϕ*_*e*_ obeying ${\mathcal{R}}({\theta }_{\varpi })$ with ${\phi }_{e}^{ > }\in {{\mathscr{D}}}_{{\omega }_{\varpi }}$ and on all eigenstates *ϕ*_*e*_ obeying ${\mathcal{R}}({\theta }_{\varpi }+\pi )$ with ${\phi }_{e}^{ > }\in {{\mathscr{D}}}_{\infty }$. This extension is maximal by construction and reads

$${\pi }_{{\theta }_{\varpi }}\left({H}_{{\omega }_{\varpi }}({{\mathbb{R}}}_{+}^{\ast })\times {H}_{{\omega }_{\varpi }}({{\mathbb{R}}}_{-}^{\ast })\right)\oplus {\pi }_{{\theta }_{\varpi }+\pi }\left({H}_{\infty }({{\mathbb{R}}}_{+}^{\ast })\times {H}_{\infty }({{\mathbb{R}}}_{-}^{\ast })\right),$$

where *π*_*θ*_ is the projector on *θ*-symmetrical functions. Since $${H}_{\varpi }({\mathbb{R}})$$ is maximal by definition, it is equal to this extension, and our classification is complete, within the assumption that there are non Rydberg eigenfunctions (one at least in the bound spectrum, one at least in the free spectrum). In such case, we define *ϖ* = (*ω*_*ϖ*_, *θ*_*ϖ*_) and our results prove that $${{\mathscr{D}}}_{\varpi }={\pi }_{{\theta }_{\varpi }}\left({{\mathscr{D}}}_{{\omega }_{\varpi }}\bigcup {\widehat{{\mathscr{D}}}}_{{\omega }_{\varpi }}\right)$$$$\oplus {\pi }_{{\theta }_{\varpi }+\pi }\left({{\mathscr{D}}}_{\infty }\bigcup {\widehat{{\mathscr{D}}}}_{\infty }\right)$$ and $${{\mathscr{S}}}_{\varpi }={{\mathscr{S}}}_{{\omega }_{\varpi }}\bigcup {{\mathscr{S}}}_{\infty }$$. ■

Let us assume now that there are no non Rydberg states. In that case, all combinations of eigenstates are orthogonal, so the self-adjoint extension is defined on ${{\mathscr{D}}}_{\infty }\bigcup {\widehat{{\mathscr{D}}}}_{\infty }$ with no constraint. It is maximal by construction, so ${H}_{\varpi }({\mathbb{R}})$ equals ${H}_{\infty }({{\mathbb{R}}}_{+}^{\ast })\times {H}_{\infty }({{\mathbb{R}}}_{-}^{\ast })$. We define *ϖ* =∞ in that situation. Note that, however, ${H}_{\infty }({\mathbb{R}})$ can be identified with ${H}_{(\omega ,\theta )}({\mathbb{R}})$ for any $\theta \in \left[0,2\pi \right.\left[\right.$ and *ω* →∞, because one can expand any eigenfunction as the sum of its *θ*-symmetrical and *θ* + *π*-symmetrical parts.

### Existence of a non Rydberg bound state

We consider a self-adjoint extension ${H}_{\varpi }({\mathbb{R}})$. We assume there is at least a non Rydberg eigenstate, otherwise *ϖ* =∞, which situation exists and has been studied above.

We can rapidly exclude the situation, where there are no non Rydberg free eigenstates. Indeed, one knows that all non Rydberg bound states’ energies belong to some set ${{\mathscr{S}}}_{{\omega }_{\varpi }}$ and that their eigenfunctions obey ${{\mathcal{R}}}_{({\theta }_{\varpi })}$, with ${\theta }_{\varpi }\in \left[0,2\pi \right.\left[\right.$; so they belong to ${{\mathscr{B}}}_{\varpi }={\pi }_{{\theta }_{\varpi }}\left({{\mathscr{B}}}_{\omega }\bigcup {\widehat{{\mathscr{B}}}}_{\omega }\right)$. Thus, ${H}_{\varpi }({\mathbb{R}})$ can be extended by ${H}_{({\omega }_{\varpi },{\theta }_{\varpi })}({\mathbb{R}})$. Therefore, ${H}_{\varpi }({\mathbb{R}})={H}_{({\omega }_{\varpi },{\theta }_{\varpi })}({\mathbb{R}})$ and there are indeed non Rydberg free eigenstates.

On the contrary, the situation with non Rydberg free eigenstates and no bound ones can not be discarded so easily. The demonstration is close to that of section ‘Existence of a bound state’.

We first study the case of a unique non Rydberg free eigenstate $\left|{\phi }_{{e}_{1}}\right\rangle$. There must be a Rydberg free one $\left|{\phi }_{{e}_{2}}\right\rangle$ with *e*_2_ ≠ *e*_1_, otherwise, ${H}_{\varpi }({\mathbb{R}})$ would not be physical. (2) reads ${\phi }_{{e}_{1}}^{ > }={\alpha }_{{k}_{1}}^{+}{F}_{{\eta }_{1}}+{\beta }_{{k}_{1}}^{+}{G}_{{\eta }_{1}}$, ${\phi }_{{e}_{1}}^{\, < \,}={\alpha }_{{k}_{1}}^{-}\widehat{{F}_{{\eta }_{1}}}+{\beta }_{{k}_{1}}^{-}\widehat{{G}_{{\eta }_{1}}}$, ${\phi }_{{e}_{2}}^{ > }={\alpha }_{{k}_{2}}^{+}{F}_{{\eta }_{1}}$ and ${\phi }_{{e}_{2}}^{\, < \,}={\alpha }_{{k}_{2}}^{-}\widehat{{F}_{{\eta }_{2}}}$.

We have found that there exists *θ*_*ϖ*_ such that ${\beta }_{{k}_{1}}^{-}={{\rm{e}}}^{i{\theta }_{\varpi }}{\beta }_{{k}_{1}}^{+}$. Then, $\langle {\phi }_{{e}_{1}}| {\phi }_{{e}_{2}}\rangle =0$ gives

$$\bar{{\beta }_{{k}_{1}}^{+}}{\alpha }_{{k}_{2}}^{+}+\bar{{\beta }_{{k}_{1}}^{-}}{\alpha }_{{k}_{2}}^{-}=0$$

so ${\alpha }_{{k}_{2}}^{-}=-{{\rm{e}}}^{i{\theta }_{\varpi }}{\alpha }_{{k}_{2}}^{+}={{\rm{e}}}^{i({\theta }_{\varpi }+\pi )}{\alpha }_{{k}_{2}}^{+}$. Since the eigenspace associated to *e*_2_ is of dimension 1 (because of the Dirichlet condition, since ${\phi }_{{e}_{2}}$ is Rydberg), one deduces that ${\phi }_{{e}_{2}}$ obeys ${\mathcal{R}}({\theta }_{\varpi }+\pi )$. Thus, from the relation above, ${\phi }_{{e}_{1}}$ obeys ${\mathcal{R}}({\theta }_{\varpi })$. Therefore, ${\alpha }_{{k}_{1}}^{+}/{\beta }_{{k}_{1}}^{+}={\alpha }_{{k}_{1}}^{-}/{\beta }_{{k}_{1}}^{-}\equiv {\zeta }_{{k}_{1}}$, one defines $\omega =-{g}_{f}({\eta }_{1})+\frac{{\zeta }_{{k}_{1}}{C}_{{\eta }_{1}}^{2}}{2{\eta }_{1}}$, then ${H}_{\varpi }({\mathbb{R}})$ can be extended by ${H}_{(\omega ,{\theta }_{\varpi })}({\mathbb{R}})$, which admits non Rydberg bound eigenstates. This is indeed contradictory and the case can be discarded. ■

Let us assume now there are two independent non Rydberg free states $\left|{\phi }_{{e}_{1}}\right\rangle$ and $\left|{\phi }_{{e}_{2}}\right\rangle$. (2) reads ${\phi }_{{e}_{1}}^{ > }={\alpha }_{{k}_{1}}^{+}{F}_{{\eta }_{1}}+{\beta }_{{k}_{1}}^{+}{G}_{{\eta }_{1}}$, ${\phi }_{{e}_{1}}^{\, < \,}={\alpha }_{{k}_{1}}^{-}\widehat{{F}_{{\eta }_{1}}}+{\beta }_{{k}_{1}}^{-}\widehat{{G}_{{\eta }_{1}}}$, ${\phi }_{{e}_{2}}^{ > }={\alpha }_{{k}_{2}}^{+}{F}_{{\eta }_{1}}+{\beta }_{{k}_{2}}^{+}{G}_{{\eta }_{2}}$ and ${\phi }_{{e}_{2}}^{\, < \,}={\alpha }_{{k}_{2}}^{-}\widehat{{F}_{{\eta }_{2}}}+{\beta }_{{k}_{2}}^{-}\widehat{{G}_{{\eta }_{2}}}$. We have already found that there exist *θ*_*ϖ*_ such that ${\beta }_{{k}_{1}}^{-}={{\rm{e}}}^{i{\theta }_{\varpi }}{\beta }_{{k}_{1}}^{+}$ and ${\beta }_{{k}_{2}}^{-}={{\rm{e}}}^{i{\theta }_{\varpi }}{\beta }_{{k}_{2}}^{+}$. We use the continuity of **j** the same way as before, constructing a state $\left|\phi \right\rangle =\alpha \left|{\phi }_{{e}_{1}}\right\rangle +\beta \left|{\phi }_{{e}_{2}}\right\rangle$ and calculating ${\mathrm{lim}}_{{\varepsilon }_{1}\to 0;{\varepsilon }_{2}\to 0}$. One applies again the independence of sinus and cosine, and skips factor $\frac{\hslash \alpha \beta }{m}$. Then, the first order of the remaining term reads

$$\left(\frac{{C}_{{\eta }_{2}}}{{\eta }_{1}{C}_{{\eta }_{1}}}\left(\bar{{\beta }_{{k}_{1}}^{+}}{\alpha }_{{k}_{2}}^{+}-\bar{{\beta }_{{k}_{1}}^{-}}{\alpha }_{{k}_{2}}^{-}\right)+\frac{{C}_{{\eta }_{1}}}{{\eta }_{2}{C}_{{\eta }_{2}}}\left(\bar{{\beta }_{{k}_{2}}^{+}}{\alpha }_{{k}_{1}}^{+}-\bar{{\beta }_{{k}_{2}}^{-}}{\alpha }_{{k}_{1}}^{-}\right)\right)\times \frac{1}{{\varepsilon }_{+}+{\varepsilon }_{2}},$$

so the existence of the limit *ε*_1_ → 0 and *ε*_2_ → 0 gives

15 $$\frac{\bar{{\zeta }_{{k}_{1}}^{+}}{C}_{{\eta }_{1}}^{2}}{{\eta }_{1}}-\frac{\bar{{\zeta }_{{k}_{1}}^{-}}{C}_{{\eta }_{1}}^{2}}{{\eta }_{1}}=\frac{{\zeta }_{{k}_{2}}^{+}{C}_{{\eta }_{2}}^{2}}{{\eta }_{2}}-\frac{{\zeta }_{{k}_{2}}^{-}{C}_{{\eta }_{2}}^{2}}{{\eta }_{2}}.$$

The second order gives $\bar{{\beta }_{{k}_{1}}^{+}}{\beta }_{{k}_{2}}^{+}=\bar{{\beta }_{{k}_{1}}^{-}}{\beta }_{{k}_{2}}^{-}$, which one already knows. The scalar product $\langle {\phi }_{{e}_{1}}| {\phi }_{{e}_{2}}\rangle$ reads

$$\langle {\phi }_{{e}_{1}}| {\phi }_{{e}_{2}}\rangle =\frac{2\lambda }{{k}_{1}^{2}-{k}_{2}^{2}}\left(\frac{{C}_{{\eta }_{2}}}{{C}_{{\eta }_{1}}}\frac{\bar{{\beta }_{{k}_{1}}^{+}}{\alpha }_{{k}_{2}}^{+}-\bar{{\beta }_{{k}_{1}}^{-}}{\alpha }_{{k}_{2}}^{-}}{2\,{\eta }_{1}}-\frac{{C}_{{\eta }_{1}}}{{C}_{{\eta }_{2}}}\times \frac{\bar{{\beta }_{{k}_{2}}^{+}}{\alpha }_{{k}_{1}}^{+}-\bar{{\beta }_{{k}_{2}}^{-}}{\alpha }_{{k}_{1}}^{-}}{2\,{\eta }_{2}}+\frac{{g}_{f}({\eta }_{1})-{g}_{f}({\eta }_{2})}{{C}_{{\eta }_{1}}{C}_{{\eta }_{2}}}\left(\bar{{\beta }_{{k}_{1}}^{+}}{\beta }_{{k}_{2}}^{+}+\bar{{\beta }_{{k}_{1}}^{-}}{\beta }_{{k}_{2}}^{-}\right)\right)$$

thus $$\langle {\phi }_{{e}_{1}}| {\phi }_{{e}_{2}}\rangle =0$$ gives

16 $$\frac{\bar{{\zeta }_{{k}_{1}}^{+}}{C}_{{\eta }_{1}}^{2}}{{\eta }_{1}}+\frac{\bar{{\zeta }_{{k}_{1}}^{-}}{C}_{{\eta }_{1}}^{2}}{{\eta }_{1}}-2{g}_{f}({\eta }_{1})=\frac{{\zeta }_{{k}_{2}}^{+}{C}_{{\eta }_{2}}^{2}}{{\eta }_{2}}+\frac{{\zeta }_{{k}_{2}}^{-}{C}_{{\eta }_{2}}^{2}}{{\eta }_{2}}-2{g}_{f}({\eta }_{2}).$$

Adding (15) and (16) proves that $({\zeta }_{{k}_{1}}^{+},{\zeta }_{{k}_{2}}^{+})$ obeys (10); thus, ${\zeta }_{{k}_{i}}^{+}$ are real and obey (11). Subtracting (15) and (16) proves that $({\zeta }_{{k}_{1}}^{-},{\zeta }_{{k}_{2}}^{-})$ obeys (10); thus, ${\zeta }_{{k}_{i}}^{-}$ are real and obey (11). Then, (16) proves that the same *ω* can be associated to all functions ${\phi }_{{k}_{1}}^{ > }$, ${\phi }_{{k}_{1}}^{\, < \,}$, ${\phi }_{{k}_{2}}^{ > }$ and ${\phi }_{{k}_{2}}^{\, < \,}$. Therefore, notwithstanding we did not establish ${\zeta }_{i}^{+}={\zeta }_{i}^{-}$, one can introduce any bound state associated to *φ*_*η*_ with *g*_b_(*η*) = −*ω*, and extend the action of ${H}_{\varpi }({\mathbb{R}})$ on these bound states, keeping the operator symmetric. This is contradictory, so the result is proved. ■

## Discussion

### Mathematical interpretation

We have determined all self-adjoint extensions. *θ*-symmetrical states obey ${\mathscr{C}}(\theta )$. So, all eigenfunctions *ϕ*_*e*_ of ${H}_{\omega ,\theta }({\mathbb{R}})$, respecting ${\mathcal{R}}(\theta )$, with ${\phi }_{e}^{ > }\in {{\mathscr{D}}}_{\omega }$, obey ${\mathscr{C}}(\theta )$ and all eigenfunctions *ϕ*_*e*_ of ${H}_{\omega ,\theta }({\mathbb{R}})$, respecting ${\mathcal{R}}(\theta +\pi )$, with ${\phi }_{e}^{ > }\in {{\mathscr{D}}}_{\infty }$, obey ${\mathscr{C}}(\theta +\pi )$. For ${H}_{\infty }({\mathbb{R}})$, ${\mathscr{C}}(\theta )$ reduces to Dirichlet conditions (all eigenfunctions obey ${\mathscr{C}}({\theta }^{{\prime} })$ for any ${\theta }^{{\prime} }\in \left[0,2\pi \right.\left[\right.$).

### The dirichlet case in one dimension

We focus on the case *ϖ* =∞ and study eigenfunctions *ϕ*_*e*_. Both attractive and repulsive case can be considered, but we will focus on the first one.

Let us consider the bound spectrum. From what precedes, *μ* in section ‘Description of a self-adjoint extension’ is entirely free. Therefore, $\{\left|{\phi }_{e}^{ > }\right\rangle ,\left|{\phi }_{e}^{ < }\right\rangle \}$ is a basis of the eigenspace *E*_*e*_ corresponding to energy *e*. This is an exceptional violation of the general result, which asserts that an energy in the bound spectrum is non degenerated in one dimensional systems. Here, the eigenspace *E*_*e*_ has dimension 2. However, examining the standard demonstration^26^, on observes that it is based on a Wronskian theorem, which can not apply here.

Another basis is composed of $\{\left|{\phi }_{e}^{+}\right\rangle ,\left|{\phi }_{e}^{-}\right\rangle \}$, the even and odd extensions on ${\mathbb{R}}$. For *ω* =∞, one observes that ${\phi }_{e}^{-}\in C({\mathbb{R}})$ for all $e\in {{\mathscr{S}}}_{\infty }\bigcup {{\mathbb{R}}}_{+}$. ${H}_{\infty }({\mathbb{R}})$, defined on these basis, is closed and therefore self-adjoint. More generally, one can use $\{\left|{\phi }_{e}^{\theta }\right\rangle ,\left|{\phi }_{e}^{\theta +\pi }\right\rangle \}$, for any $\theta \in \left[0,2\pi \right.\left[\right.$.

## Physical applications

We study different possible extensions of this work to real physical situations.

### The hydrogenate case in three dimension

Let us focus on the case ${\mathbb{D}}={{\mathbb{R}}}^{3}$, using the mapping *Φ*(*r*) = *ϕ*(*r*)/*r*, where *ϕ* is the one-dimensional solution and *Φ* the radial part of the three-dimensional wavefunction. We will only consider the attractive case here.

Let us connect our parametrization *ω* with that of ref. ^14^, which parameter is written *α*. We will show the connection for bound states only, but this can be done for all states. The first order expansion of any state *ϕ*_*e*_ with *e* < 0 reads

$${\phi }_{e}(x)=a+\lambda axln\,(| \,\lambda \,| x)+bx;$$

this expression holds both in attractive and repulsive cases. *ω* can be expressed in terms of *b*/*a*, which reads

$$\omega =\frac{1}{| \,\lambda \,| }\left(\frac{b}{a}-\lambda \right).$$

In ref. ^14^, where *λ* reads *γ*, one finds parameters *ϕ*_0_ = *a* and *ϕ*_1_ = *b*, so one gets

$$\alpha =\frac{1}{4\pi }\frac{b}{a}\quad \;\iff \;\quad \omega =\frac{1}{| \,\lambda \,| }(4\pi \alpha -\lambda ).$$

As it is well known^27^, for *L* > 0, the solutions of the Schrödinger equation which do not cancel at *r* = 0 do not belong to ${L}^{2}({{\mathbb{R}}}^{3})$ and must therefore be discarded. On the contrary, that, corresponding to the case *L* = 0, belong to ${L}^{2}({{\mathbb{R}}}^{3})$ (all *g*_*η*_ solutions, which diverge at *r* →∞ are excluded from this discussion). This is the reason why the *L* ≠ 0 subspaces appearing in (2.1.13) of ref. ^14^ have no parametrization, contrary to the *L* = 0 one.

This helps us interpreting what these authors mean by  ≪*H*_*γ*,*α*,*y*_ describes the Coulomb interaction plus an additional point interaction ≫ : the eigenfunctions for *α* <∞ are divergent eigenfunctions and not physical, although they belong to ${L}^{2}({{\mathbb{R}}}^{3})$, so they do not describe the physical Coulomb interaction. Most authors have similarly assumed that the only admissible Coulomb bound states are the Rydberg ones, given by the Laguerre polynomial

$$\Phi (r)=\sqrt{\frac{2\lambda }{{n}^{3/2}}}{{\rm{e}}}^{-r}{L}_{n}^{{\prime} }(2r)$$

with a specific normalization (assuming that the spherical function reads $1/\sqrt{4\pi }$ for kinetic momentum *L* = 0). This solution exactly corresponds to the *ω* =∞ Dirichlet case, which is also the *α* =∞ one.

Actually, no fundamental principle of quantum mechanics justifies discarding solutions that diverge for *r* → 0, since the probability ∫ǀΦ(*r*)ǀ^2^*r*^2^*dr* is finite (in the basic meaning "not infinite”). However, experimental evidences, from the original Rydberg spectrum, are in excellent agreement with this assumption. We find that experimental data^28^ are only compatible with ǀ*ω*ǀ > 27779. We have simply compared the ratio $\frac{{E}_{2}-{E}_{m}}{{E}_{2}-{E}_{n}}$, for several (*m*, *n*) couples, as determined from these data, with that calculated from the exact values of ${{\mathscr{S}}}_{\omega }$. Actually, (*m*, *n*) = (5, 3) gives the highest (best) limit of possible values for *ω*.

Based on these physical grounds, we will follow the common choice and, dealing with the case $D={{\mathbb{R}}}^{3}$, discard all divergent wavefunctions, therefore reducing the parameter range to *ω* =∞, the self-adjoint extension corresponding to Dirichlet solutions. We can justify this choice, from a mathematical point of view, by reminding that the deficiency coefficient of $H({{\mathbb{R}}}^{3})$ is zero. We will discuss this point further on.

### Explicit spectra for a semi-infinite line

**Figure 6:**
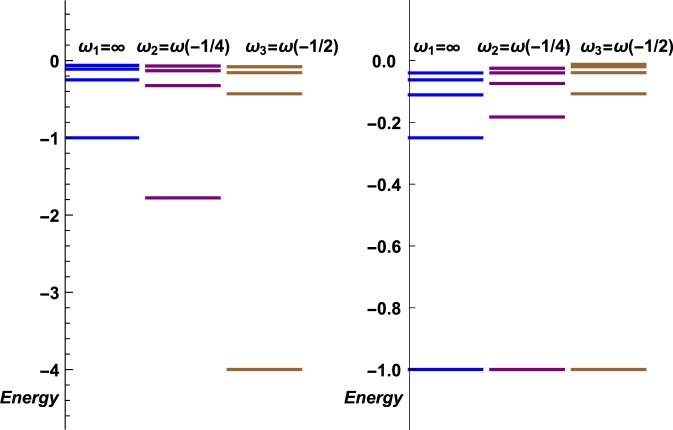
Spectra for *ω*_*i*_ (*i* = 1, 2, 3) defined in the text. On the left, we show the absolute values, on the right, we normalize energies so that the lowest energy is  −1. The variation of *E*_*n*+1_ −*E*_*n*_, when *n* is increased, is steeper for *ω*_3_.

The calculated spectra ${{\mathscr{S}}}_{\omega }({{\mathbb{R}}}_{+}^{\ast })$ vary significantly, for different values of *ω*. We show three of them in Fig. 6, corresponding to *ω*_1_ =∞ (Rydberg spectrum), *ω*_2_ = *ω*( −1/4) ≈ 2.3 and *ω*_3_ = *ω*( −1/2) ≈ −0.27 (close to the Neumann case). As already pointed out, in any one-dimensional system the experimental determination of this spectra would allow that of the limit $\phi {\prime} ({0}^{+})/\phi ({0}^{+})$ in the vicinity of the charge defect. Turning back to the one-dimensional attractive case, defined in ${\mathbb{D}}={{\mathbb{R}}}_{+}^{\ast }$, one observes that the previous physical arguments used in the three-dimensional case cannot apply, because no wavefunction is ever diverging. Therefore, one must consider all parameters, $\omega \in \left[-\infty ,\infty \right]$ in the attractive case, $\omega \in \left[-\infty ,2{\gamma }_{E}\right]$ in the repulsive one.

The determination of *ω* is highly system dependent. If any experimental spectrum, close enough to this case, can be measured in the future with high enough precision, like that of a one-dimensional quantum wire (like a carbon nanotube) with a charge defect at one extremity or an hydrogen atom in very intense magnetic field, then we argue that the limit condition $\phi {\prime} (0)/\phi (0)$, at that extremity, will be determined by examining the sequence of energies *E*_1_ < *E*_2_ < *E*_3_... and in particular the sequence of their ratio.

### Regularization of the potential

We consider here the regularized potential ${V}_{\varepsilon }=\left.\lambda /\sqrt{{x}^{2}+{\varepsilon }^{2}}\right)$ in the attractive case, with ${\mathbb{D}}={\mathbb{R}}$. This is a way to address the 1 + *ε*-dimensional case, since this potential describes the situation where the charge is lightly displaced from axis ${\mathbb{R}}$ in the 3-dimensional space. When *ε* → 0, it converges towards the Coulomb potential, *V*_*ε*_ → *V*. We focus on the negative (bound) spectrum of the corresponding Hamiltonian ${H}_{\varepsilon }({\mathbb{R}})$, which is self-adjoint.

**Figure 7:**
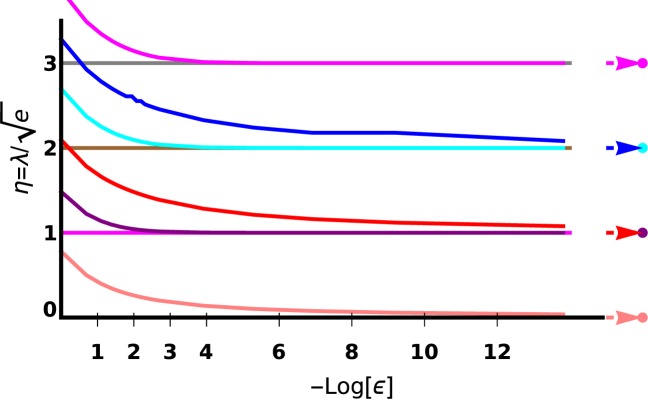
First energies of $\frac{{p}^{2}}{2m}+{V}_{\varepsilon }$ (${e}_{\frac{1}{2}}^{\varepsilon }$, ${e}_{1}^{\varepsilon }$, ${e}_{\frac{3}{2}}^{\varepsilon }$, ${e}_{2}^{\varepsilon }$, ${e}_{\frac{5}{2}}^{\varepsilon }$ and ${e}_{3}^{\varepsilon }$ from bottom to top) versus $ln\,(1/\varepsilon )$ in dimensionless *y*-scale. The asymptotic limit is indicated by an arrow on the right, for each curve and by the horizontal straight lines.

This spectrum is found discrete and non degenerate  ∀ *ε* ≠ 0. In this case, all eigenfunctions are orthogonal and form a complete basis, because they obey to $H({{\mathbb{R}}}^{3})$, which is self-adjoint, as explained before. They separate into two groups, odd functions ${\chi }_{2p}^{\varepsilon }$ with $p\in {{\mathbb{N}}}^{\ast }$, and even ones ${\chi }_{2p+1}^{\varepsilon }$ with $p\in {\mathbb{N}}$. We will note ${e}_{p}^{\varepsilon }$ the energies corresponding to odd solutions, and ${e}_{p+\frac{1}{2}}^{\varepsilon }$ that of even solutions. Figure 7 shows the first (smallest) energies as a function of $ln\,1/\varepsilon$. When *ε* → 0, even wavefunctions ${\chi }_{2p}^{\varepsilon }\to {\chi }_{2p}^{0}={\phi }_{-{\lambda }^{2}/(4{p}^{2})}$ while their energy rapidly reaches  −*λ*^2^/(4*p*^2^) the corresponding Rydberg energy. Odd ones also ${\chi }_{2p+1}^{\varepsilon }\to {\phi }_{-{\lambda }^{2}/(4{p}^{2})}$ while their energy reaches  −*λ*^2^/(4*p*^2^) the Rydberg energy. This is conform with the 2-degeneracy that is proved in the case *ϖ* = {∞^+^,∞^−^}, which shows that ${H}_{\varepsilon }({\mathbb{R}})\to {H}_{\infty }({\mathbb{R}})$. An odd eigenfunction seems to be converging towards a zero energy eigenfunction, but it vanishes as *ε* → 0, in conformity with our discussion about these functions.

## Spectral theorem

We discuss the way one should write the spectral theorem, in the case of incompatible self-adjoint extensions.

### Spectral theorem in ${{\mathbb{R}}}_{+}^{\ast }$

For each value *ω*, $${H}_{\omega }({{\mathbb{R}}}_{+}^{\ast })$$ is self-adjoint, so the spectral theorem is valid. Therefore, any function $${\psi }_{i}\in {L}^{2}({{\mathbb{R}}}_{+}^{\ast })$$ can be developed on the basis $${{\mathscr{B}}}_{\omega }({{\mathbb{R}}}_{+}^{\ast })\bigcup $$$${{\mathscr{F}}}_{\omega }({{\mathbb{R}}}_{+}^{\ast })$$

$${\psi }_{i}(x)={\sum }_{\eta \in {{\mathscr{S}}}_{\omega }}{b}_{k}^{i}{\varphi }_{k}(kx)+{\int }_{{{\mathbb{R}}}_{+}}{c}_{k}^{i}{\Psi }_{k}(kx)\frac{dk}{\pi }\,\mathrm{with}\,{b}_{k}^{i}=\langle {\varphi }_{k}| {\psi }_{i}\rangle \,\mathrm{and}\,{c}_{k}^{i}=\langle {\Psi }_{k}| {\psi }_{i}\rangle .$$

endeqnarray* For *ω* =∞ and *λ* < 0 (attractive case), this formula is equivalent to Eq. 19.171, in ref. ^26^ with a different normalization (we preferred to use *k* parameter, rather than *E*). We have checked this formula numerically on several examples, $x\mapsto {{\rm{e}}}^{-{x}^{2}}$, $x\mapsto x\,{{\rm{e}}}^{-{x}^{2}}$, etc. One can, in particular, expand a function *φ*_*k*_, with $\omega \left(\frac{\lambda }{2k}\right)\ne {\omega }_{1}$ on ${{\mathscr{B}}}_{{\omega }_{1}}({{\mathbb{R}}}_{+}^{\ast })\bigcup {{\mathscr{F}}}_{{\omega }_{1}}({{\mathbb{R}}}_{+}^{\ast })$, which we have done for functions *ψ*_0_ = *φ*_−*λ*_ (setting ${\omega }_{0}=\omega \left(-\frac{1}{2}\right)$) or ${\psi }_{0}={\varphi }_{-\frac{\lambda }{3}}$ (setting ${\omega }_{0}=\omega \left(-\frac{3}{2}\right)$), while choosing *ω*_1_ =∞.

*ω* = *ω*_0_ or *ω* = *ω*_1_, is well defined by this expansion, writing for instance

$${H}_{{\omega }_{1}}({{\mathbb{R}}}_{+}^{\ast })\left|{\psi }_{0}\right\rangle =-{\sum }_{-{k}_{1}^{2}\in {{\mathscr{S}}}_{{\omega }_{1}}}{k}_{1}^{2}{b}_{{k}_{1}}^{0}{\varphi }_{{k}_{1}}({k}_{1}x)+{\int }_{{{\mathbb{R}}}_{+}}{k}_{1}^{2}{c}_{{k}_{1}}^{0}{\Psi }_{{k}_{1}}({k}_{1}x)\frac{d{k}_{1}}{\pi }.$$

This result differs from ${H}_{{\omega }_{0}}({{\mathbb{R}}}_{+}^{\ast })\left|{\psi }_{0}\right\rangle$, which reads

$${H}_{{\omega }_{0}}({{\mathbb{R}}}_{+}^{\ast })\left|{\psi }_{0}\right\rangle =-{k}_{0}^{2}\left|{\psi }_{0}\right\rangle =-{\sum }_{-{k}_{1}^{2}\in {{\mathscr{S}}}_{{\omega }_{1}}}{k}_{0}^{2}{b}_{{k}_{1}}^{0}{\varphi }_{{k}_{1}}({k}_{1}x)-{\int }_{{{\mathbb{R}}}_{+}}{k}_{0}^{2}{c}_{{k}_{1}}^{0}{\Psi }_{{k}_{1}}({k}_{1}x)\frac{d{k}_{1}}{\pi }.$$

Finally, one should be aware that, as a formal derivative operator, the action of $H({{\mathbb{R}}}_{+}^{\ast })$ on *ψ*_0_ is well defined. In particular, one is interested by its action on eigenfunctions *ϕ*_*e*_. One eventually finds

$$H({{\mathbb{R}}}_{+}^{\ast })\left|{\phi }_{e}\right\rangle =e\left|{\phi }_{e}\right\rangle $$

which means that $H({{\mathbb{R}}}_{+}^{\ast })$ acts on *ψ*_*e*_ as ${H}_{\omega }({{\mathbb{R}}}_{+}^{\ast })$ with *ω* = *ω*(*e*), the index of energy *e*. However, $H({{\mathbb{R}}}_{+}^{\ast })$ is not a *good* operator, because it does not correspond to the same self-adjoint extension, for each state.

Technically, the last result can be understood as follows: *d*/*dx* does not commute with ∫*dk* in the former development. Indeed, when the derivation is performed **inside** the integral, it produces a factor *η* ∝ 1/*k* which makes it improper.

This analysis is common with that, which can be made for *H* = −*d*^2^/*dx*^2^; the divergence of the Coulomb potential is not entirely responsible of the loss of self-adjointness.

### Spectral theorem in ${\mathbb{R}}$

The spectral theorem in ${\mathbb{R}}$ can be formulated after that in ${{\mathbb{R}}}_{+}^{\ast }$. Each *θ*-symmetrical and *θ* + *π*-symmetrical part of any function can be expanded separately. Considering ${H}_{\varpi }({\mathbb{R}})$, with *ϖ* = (*ω*, *θ*), any function $\phi \in {L}^{2}({\mathbb{R}})$ expands into *ϕ* = *ϕ*^*θ*^ + *ϕ*^*θ*+*π*^. Then *ϕ*^*θ*^ expands in ${{\mathscr{B}}}_{\omega }({\mathbb{R}})\bigcup {{\mathscr{F}}}_{\omega }({\mathbb{R}})$ exactly as *ϕ*^*θ*>^ in ${{\mathscr{B}}}_{\omega }({{\mathbb{R}}}_{+}^{\ast })\bigcup {{\mathscr{F}}}_{\omega }({{\mathbb{R}}}_{+}^{\ast })$ but for a supplementary factor $1/\sqrt{2}$: one should take the expansion calculated for ${\mathbb{D}}={{\mathbb{R}}}_{+}^{\ast }$ and allow $x\in {\mathbb{R}}$; similarly *ϕ*^*θ*+*π*^ expands in ${{\mathscr{B}}}_{\infty }({\mathbb{R}})\bigcup {{\mathscr{F}}}_{\infty }({\mathbb{R}})$ exactly as *ϕ*^*θ*+*π*>^ in ${{\mathscr{B}}}_{\infty }({{\mathbb{R}}}_{+}^{\ast })\bigcup {{\mathscr{F}}}_{\infty }({{\mathbb{R}}}_{+}^{\ast })$ but for a supplementary factor $1/\sqrt{2}$.

It applies also in the particular case *ϖ* =∞, choosing any arbitrary *θ*. In this case, one can also write *f* = *f*^>^ + *f*^<^ (where *f*^>^ extends in ${{\mathbb{R}}}_{-}^{\ast }$ as zero and *f*^<^ extends in ${{\mathbb{R}}}_{+}^{\ast }$ as zero). *f*^>^ expands in ${{\mathscr{B}}}_{\infty }({{\mathbb{R}}}_{+}^{\ast })\bigcup {{\mathscr{F}}}_{\infty }({{\mathbb{R}}}_{+}^{\ast })$ and *f*^<^ expands in ${{\mathscr{B}}}_{\infty }({{\mathbb{R}}}_{-}^{\ast })\bigcup {{\mathscr{F}}}_{\infty }({{\mathbb{R}}}_{-}^{\ast })$. This is the right place to observe that *μ*, defined in in section ‘Description of a self-adjoint extension’, is not determinate in this particular case. One can indeed choose *μ* = 0 (i.e. *f* = *f*^>^) or *μ* =∞ (i.e. *f* = *f*^<^). We discuss this supplementary degree of freedom further.

## Topological classification of the extension parameter space

### Structure for ${\mathbb{D}}={{\mathbb{R}}}_{+}^{\ast }$ in the repulsive case

The structure of the order parameter seems to be equivalent to the interval $\left[-\infty ,2{\gamma }_{E}\right]$ in the repulsive case, which is topologically equivalent to interval $\left[0,1\right]$. This is notwithstanding the special case ${H}_{2{\gamma }_{E}}({{\mathbb{R}}}_{+}^{\ast })$, which we found for the zero energy. This case corresponds to *ω* = 2*γ*_E_, but, what should now be pointed out is that the regular limit *ω* → 2*γ*_E_, which can be constructed, using *g*_*η*_, does not exist. One finds indeed that eigenfunction *g*_*η*_ tends to a singular distribution with {0} support. Looking for such a solution, one substitutes again ${\varphi }_{0}={\sum }_{n=0}^{\infty }{a}_{n}{\delta }^{(n)}$ in (1). When *η* →∞, one finds that all coefficients *a*_*n*_ are zero.

This indicates that the right boundary of $\left[-\infty ,2{\gamma }_{E}\right]$ is apart, one should write, instead, $\left[-\infty ,2{\gamma }_{E}\right]\bigcup \{2{\gamma }_{E}\}$ and draw  to characterize this space, which is topologically equivalent to $({\mathbb{R}},+\infty )$.

### *U*(1) structure for ${\mathbb{D}}={{\mathbb{R}}}_{+}^{\ast }$ in the attractive case

**Figure 8:**
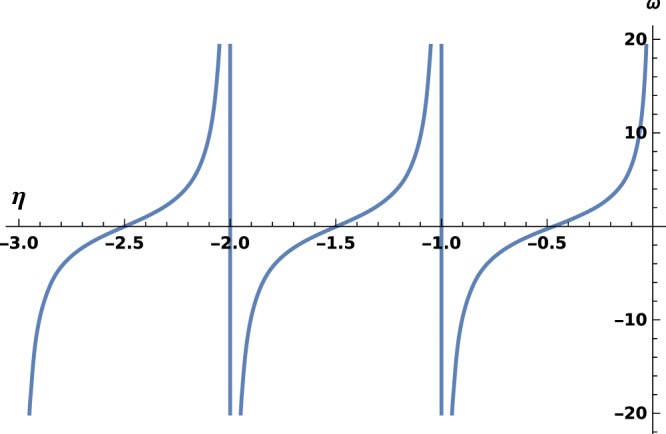
*ω* versus *η* in the attractive case.

Let us observe on Fig. 8 the curve of index *ω*(*η*) in the attractive case. It is not periodic, but there is an infinity of vertical asymptotes at positions *η* = −*n*, $n\in {{\mathbb{N}}}^{\ast }$. Any interval [ −*n* −1, −*n*], for $n\in {\mathbb{N}}$, covers all indices *ω*. In other words, any eigenfunction ${\varphi }_{\eta }\in {L}^{2}({{\mathbb{R}}}_{+}^{\ast })$, with index *η*, belongs to the bound spectrum of ${H}_{\omega }({{\mathbb{R}}}_{+}^{\ast })$, where *ω*(*η*) is determined by this curve. *ω* → −∞ and *ω* →∞ are identified (to the Rydberg solutions), which proves that the set of all extensions of $H({{\mathbb{R}}}_{+}^{\ast })$ is mapped on a space, which is topologically equivalent to the circle *U*(1).

### Structure for ${\mathbb{D}}={\mathbb{R}}$

It is worth pointing out that, although the space of extension parameter is reduced, as a consequence of the continuity condition at *x* = 0, we get the same deficiency coefficients as Oliveira *et al*.^12^ in that ${\mathbb{D}}={\mathbb{R}}$ case, which are (2, 2).

**Figure 9:**
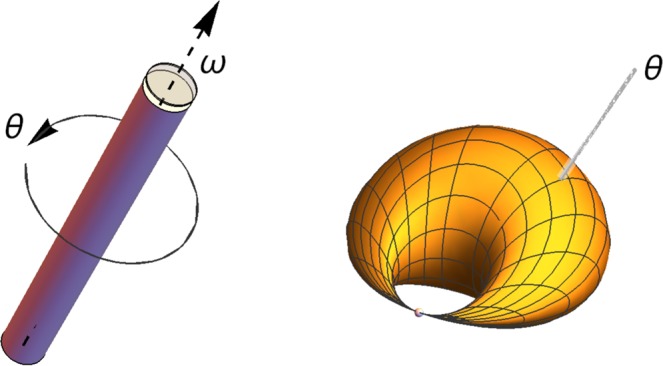
Representation of the order parameter space in the repulsive case (left) or attractive case (right). Left are represented the *ω* = 2*γ*_E_ closing circle, the *ω* axis (which is supposed to vary from  −∞ to 2*γ*_E_) and the gauge parameter *θ*. Right is represented the *ω* = ±∞ point at the strangling point and a *θ*-circle is pointed out: all orthogonal lines to this circle vary with *ω*.

Since there is no Rydberg state in the repulsive case, the structure due to parameters (*ω*, *θ*) is very simple, it is an infinite cylinder $({\mathbb{R}},+\infty )\times \left[0,2\pi \right]$, with a closed boundary at one side, as represented in Fig. 9. The structure in the attractive case is more like a torus, with a strangling, that is a singular point of infinitely small narrowness, corresponding to *ω* = ±∞, as seen on Fig. 9.

The *θ*-symmetry introduces a phase factor  ± e^i*θ*^ when a particle passes *x* = 0. Factor e^i*θ*^ is arbitrary but identical for all states associated to ${H}_{(\omega ,\theta )}({\mathbb{R}})$, similarly to standard gauge symmetry.

## Conclusion

The one-dimensional Schrödinger equation with a Coulomb *λ*/|*x*| potential brings unusual difficulties, for the physical interpretation of its solutions. Indeed, the corresponding hamiltonians $H({{\mathbb{R}}}_{+}^{\ast })$ and $H({\mathbb{R}})$ admit an infinity of self-adjoint extensions, classified by a real parameter *ω*. In the case of $H({{\mathbb{R}}}_{+}^{\ast })$ with an attractive Coulomb potential, *ω* is defined in the space ${\mathbb{R}}$ where  −∞ is identified with ∞; this space is topologically equivalent to the circle *U*(1). In the case of $H({{\mathbb{R}}}_{+}^{\ast })$ with a repulsive Coulomb potential, *ω* is defined in $\left[-\infty ,2{\gamma }_{E}\right]$. In both cases, parameter *ω* must be chosen according to the limit $\frac{\partial \phi (x)}{| \,\lambda \,| \partial x}/\phi (x)\pm ln\,(| \,\lambda \,| x)$ when *x* → 0, where  ±  is the sign of *λ*. In the attractive case, the particular value *ω* =∞ brings the Dirichlet solutions, which obey *ϕ*(0) = 0 and correspond to the standard Rydberg spectrum, while the other spectra are unusual and have never been observed yet. In the repulsive case, the particular value *ω* = 2*γ*_E_ gives a continuous spectrum *R*_+_, the zero energy eigenfunction of which is bounded.

In the case of $H({\mathbb{R}})$, physical constraints yield a phase gauge *θ*, which describes the discontinuity of wavefunctions at *x* = 0. If the Coulomb potential is attractive, two situations may occur: either one finds two separate spectra, the eigenstates of which are orthogonal and obey, respectively, ${\mathcal{R}}(\theta )$ and ${\mathcal{R}}\left(\theta +\frac{\pi }{2}\right)$ symmetry; or the spectrum is the standard Rydberg one, with an exceptional 2-degeneracy of all eigenfunctions. We did not study the repulsive case here, but we induce that there is also a supplementary symmetry ${\mathcal{R}}(\theta )$, giving the representation sketched in Fig. 9 (left).

This study brings up new considerations about quantum physics: in order to conciliate the classification of $H({{\mathbb{R}}}_{+}^{\ast })$ and $H({{\mathbb{R}}}^{3})$ with standard experimental measures of the hydrogen electronic energy levels, one has to discard all **divergent** wavefunctions, but we could not justify this choice. So we suggest to add a postulate in quantum physics, stipulating that no divergent wavefunction can be admitted, in other words all wavefunctions are bounded. Indeed, this would give an explanation why one never observes any physical states with *ω* ≠∞.

This work shows that one must be very careful when using the spectral theorem for an unbounded hamiltonian. At a time when theoretical physics research includes new and mathematically unexpected objects (like complex eigenvalues for hamiltonians, skyrmions, Majorana fermions), advanced studies of non self-adjoint hamiltonians are necessary, and, what seemed old-fashioned physics reveals an essential source of inspiration and comprehension, to determinate whether a self-adjoint extension is valid or not.

## Supplementary information

Incompatible Coulomb hamiltonian extensions: Appendix.
