# Author Correction: Incompatible Coulomb hamiltonian extensions

**DOI:** 10.1038/s41598-020-68526-w

**Published:** 2020-07-02

**Authors:** G. Abramovici

**Affiliations:** Université Paris-Saclay, CNRS, Laboratoire de Physique Des Solides, 91405 Orsay, France

Correction to: *Scientific Reports* https://doi.org/10.1038/s41598-020-62144-2, published online 29 April 2020

This Article contains errors in Figures 1 and 4. In Figure 1, some of the curves are incorrect due to a change in the behaviour of the function « HarmonicNumber » in Mathematica since the figure was created in 2015. In Figure 4, the curves have not been normalised as stated in the legend, leading to scaling change in the y-axis. The correct Figures 1 and 4 appear below as Figures 1 and 2 respectively.

**Figure 1:**
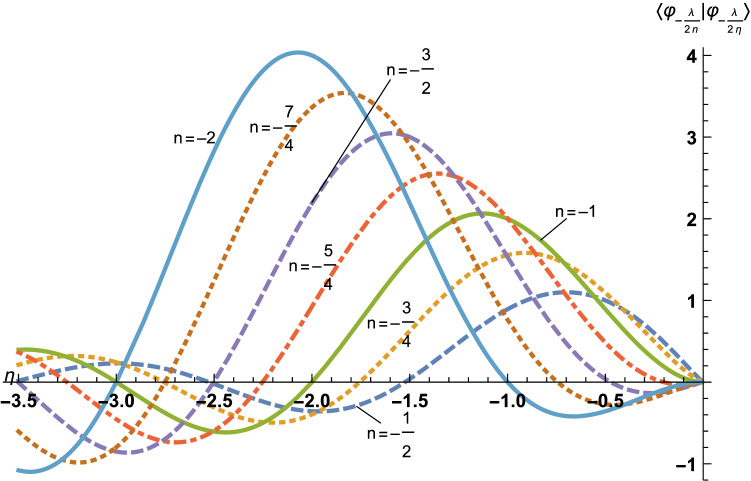
Here are the curves $\eta \mapsto \langle {\varphi }_{-\frac{\lambda }{2n}}| {\varphi }_{-\frac{\lambda }{2\eta }}\rangle$, for *n* =  − 1/2 (dashed line), *n* =  − 3/4 (dotted line), *n* =  − 1 (plain line), *n* =  − 5/4 (dot-dashed line), *n* =  − 3/2 (dashed line), *n* =  − 7/4 (dotted line) and *n* =  − 2 (plain line). The zeros of each curve read $\eta = \frac{\lambda }{{2\sqrt { - e} }}$ where $e \in {\mathscr{S}}_{\omega }$, with *ω* =  − *g*_b_(*n*), as explained further on. The curves seem to form pairs corresponding to (*n*, *n* + 1), in particular, one could believe that each pair intersects on the *η*-axis (abscissa), but this is wrong, except for (*n*, *n* + 1) = (− 2, − 1) which correspond to the same Rydberg set ${\mathscr{S}}_{\infty }$. All the other intersections are only close to zero, so that, indeed, ${\mathscr{S}}_{{ - g_{b} (n)}} \ne {\mathscr{S}}_{{ - g_{b} (n + 1)}}$. *η* = *n* is missing, because $\langle \varphi_{{ - \frac{\lambda }{2n}}} |\varphi_{{ - \frac{\lambda }{2n}}} \rangle \ne 0$.

**Figure 2:**
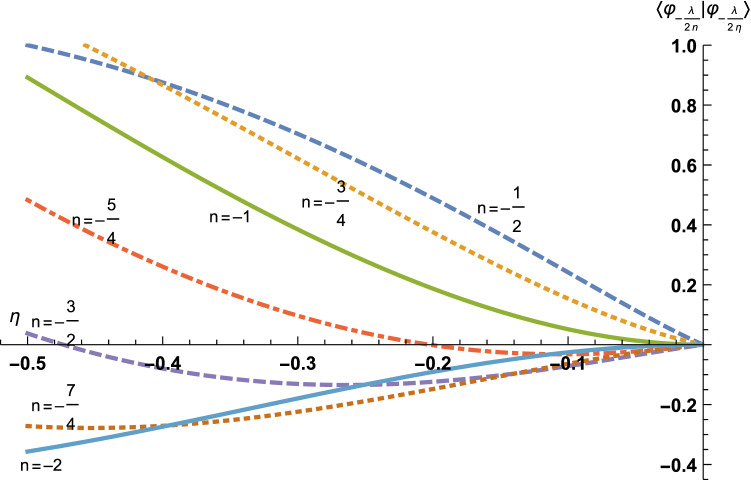
Here is a zoom of Fig. 1 in the interval $\left[- \,\frac{1}{2},0 \right]$.

