# Supplementary material for: Incompatible Coulomb hamiltonian extensions
Source: Sci Rep. 2020 Apr 29;10:7280. doi: 10.1038/s41598-020-62144-2 (PMC7190704; doi:10.1038/s41598-020-62144-2)
Supplement: Supplementary file 1 — Incompatible Coulomb hamiltonian extensions: Appendix. [file 41598_2020_62144_MOESM1_ESM.pdf]

Incompatible Coulomb hamiltonian extensions: Appendix

G. Abramovici

Université Paris-Saclay, CNRS, Laboratoire de Physique des Solides, 91405, Orsay, France

abramovici@lps.u-psud.fr

1 Overlappings

Some overlappings have been calculated in Ref. 1. We give here a general derivation of all scalar products. Let us first consider the case of a bound-unbound product $\langle\varphi_{k_1}|\Psi_{k_2}\rangle$. One has

$$\begin{aligned}\int_0^\infty dx\,\varphi_{k_1}(x)\frac{\partial^2\Psi_{k_2}}{\partial x^2}(x)-\Psi_{k_2}(x)\frac{\partial^2\varphi_{k_1}}{\partial x^2}(x) &= \left[\varphi_{k_1}(x)\frac{\partial\Psi_{k_2}}{\partial x}(x)-\Psi_{k_2}(x)\frac{\partial\varphi_{k_1}}{\partial x}(x)\right]_{0^+}^\infty\\ \text{substituting (1) of the main part for }\Psi\text{ or }\varphi\text{, one gets} &= -({k_1}^2+{k_2}^2)\int_0^\infty dx\;\varphi_{k_1}(x)\Psi_{k_2}(x)\end{aligned}$$

which leads to eq. (7) of the main part. The case of a bound-bound product $\langle\varphi_{k_1}|\varphi_{k_2}\rangle$ is similar. One has

$$\begin{aligned}\int_0^\infty dx\varphi_{k_1}(x)\frac{\partial^2\varphi_{k_2}}{\partial x^2}(x)-\varphi_{k_2}(x)\frac{\partial^2\varphi_{k_1}}{\partial x^2}(x) &= \left[\varphi_{k_1}(x)\frac{\partial\varphi_{k_2}}{\partial x}(x)-\varphi_{k_2}(x)\frac{\partial\varphi_{k_1}}{\partial x}(x)\right]_{0^+}^\infty\\ \text{substituting (1) of the main part, one gets} &= ({k_2}^2-{k_1}^2)\int_0^\infty dx\;\varphi_{k_1}(x)\varphi_{k_2}(x)\end{aligned}$$

which leads to eq. (4) of the main part. We verify the normalization of free states by the same way. One has

$$\begin{aligned}\int_0^\infty dx\Psi_{k_1}(x)\frac{\partial^2\Psi_{k_2}}{\partial x^2}(x)-\Psi_{k_2}(x)\frac{\partial^2\Psi_{k_1}}{\partial x^2}(x) &= \left[\Psi_{k_1}(x)\frac{\partial\Psi_{k_2}}{\partial x}(x)-\Psi_{k_2}(x)\frac{\partial\Psi_{k_1}}{\partial x}(x)\right]_{0^+}^\infty\\ \text{substituting (1) of the main part, one gets} &= ({k_1}^2-{k_2}^2)\int_0^\infty dx\;\Psi_{k_1}(x)\Psi_{k_2}(x)i\;.\end{aligned}$$

While, in previous calculations, the only non zero contribution of the [ ] lies at $x=0^+$, here, both this boundary and the infinite one contributes. We have not given in (9) the detailed calculation of this last contribution. Let us replace ∞ by L and take $L\to\infty$ afterwards. For $\langle F_{\eta_1}|F_{\eta_2}\rangle$ and $\langle G_{\eta_1}|G_{\eta_2}\rangle$, one finds a main contribution

$$\begin{aligned}m_{\eta_1\eta_2} &\equiv \sin\big(k_1L-k_2L-\eta_1\ln(2k_1L)+\eta_2\ln(2k_2L)+\Gamma(1+\mathrm{i}\eta_1)-\Gamma(1+\mathrm{i}\eta_2)\big)\\ &= \sin((k_1-k_2)A_{\eta_1\eta_2})\cos(s_{\eta_1\eta_2})+\cos((k_1-k_2)A_{\eta_1\eta_2})\sin(s_{\eta_1\eta_2})\;,\end{aligned}$$

with $A_{\eta_1\eta_2}{=}L+2\frac{\eta_1\eta_2}{\lambda}\ln(|\lambda|L)$ and $s_{\eta_1\eta_2}{=}\eta_1\ln|\eta_1|-\eta_2\ln|\eta_2|+\Gamma(1+\mathrm{i}\eta_1)\ -\Gamma(1+\mathrm{i}\eta_2)$. The other contributions expand as power of $1/L$ and are suppressed when $L\to\infty$. Similarly, $\langle G_{\eta_1}|F_{\eta_2}\rangle$ gives a contribution

$$\begin{aligned}n_{\eta_1\eta_2} &\equiv \cos\big(k_1L-k_2L-\eta_1\ln(2k_1L)+\eta_2\ln(2k_2L)+\Gamma(1+\mathrm{i}\eta_1)-\Gamma(1+\mathrm{i}\eta_2)\big)\\ &= \cos((k_1-k_2)A_{\eta_1\eta_2})\cos(s_{\eta_1\eta_2})-\sin((k_1-k_2)A_{\eta_1\eta_2})\sin(s_{\eta_1\eta_2})\;,\end{aligned}$$

while $\langle F_{\eta_1}|G_{\eta_2}\rangle$ gives $-n_{\eta_1\eta_2}$. The other contribution also cancel as powers of $1/L$. Note that $A_{\eta_1\eta_2}\to\infty$ when $L\to\infty$, whatever η_1 and η_2, so we will write A for short.

Eventually, taking into account all factors $\alpha^\omega_{\eta_1}$, $\alpha^\omega_{\eta_2}$, $\beta^\omega_{\eta_1}$ and $\beta^\omega_{\eta_2}$ and $1/({k_1}^2-{k_2}^2)$, one finds exactly three kinds of contribution,

$$(\alpha^\omega_{\eta_1}\alpha^\omega_{\eta_2}+\beta^\omega_{\eta_1}\beta^\omega_{\eta_2})\sin((k_1-k_2)A)/(k_1-k_2)\;,\qquad z(k_1,k_2)\sin(A(k_1-k_2))\quad\text{and}\quad z(k_1,k_2)\cos(A(k_1-k_2))$$

where z are non given continuous functions, while the first kind occurs only once and may be rewritten $\big((\alpha^\omega_{\eta_1})^2+(\beta^\omega_{\eta_1})^2\big)\sin((k_1-k_2)A)/(k_1-k_2)$, since the difference is of the second kind (note that all these functions z arise from a cancellation of the k_1-k_2 factor in the denominator with a similar factor in the numerator arising from Young-Taylor expansion). One must take $|\alpha^\omega_{\eta_1}|^2+|\beta^\omega_{\eta_1}|^2=1$ to get generalized orthonormality. Taking into account the dk/π integration factor (which means dividing by π), on finds $\sin((k_1-k_2)A)/(k_1-k_2)\to\delta(k_1-k_2)$ when $A\to\infty$. Both $\sin(A(k_1-k_2))\to0$ and $\cos(A(k_1-k_2))\to0$ as $L\to\infty$, so all the contribution of the second and third kind are suppressed at this limit. Thus our calculations are proved.■

2 Decomposition of a function

For all $\theta \in [0, 2\pi[$, any function f, $\mathbb{R} \to \mathbb{C}$, can be decomposed into θ-symmetrical and $\theta+\pi$-symmetrical parts, $f = f_\theta + f_{\theta+\pi}$. The demonstration is similar to that of the decomposition into even and odd parts. We write $f^>$ the restriction of f on $\mathbb{R}_+^*$ and $f^<$ that on $\mathbb{R}_-^*$. Then, one finds

$$f_\theta(x) = \begin{cases} f_\theta^>(x) & \forall x > 0\,; \\ f_\theta^<(x) & \forall x < 0\,; \end{cases} \qquad \text{with} \qquad f_\theta^> = \frac{f^> + \mathrm{e}^{\mathbb{i}\theta}\,\breve{f^<}}{2}\,; f_\theta^< = \frac{f^< + \mathrm{e}^{-\mathbb{i}\theta}\,\breve{f^>}}{2}\,;$$

$$f_{\theta+\pi}(x) = \begin{cases} f_{\theta+\pi}^>(x) & \forall x > 0\,; \\ f_{\theta+\pi}^<(x) & \forall x < 0\,; \end{cases} \qquad \text{with} \qquad f_{\theta+\pi}^> = \frac{f^> - \mathrm{e}^{\mathbb{i}\theta}\,\breve{f^<}}{2}\,; f_{\theta+\pi}^< = \frac{f^< - \mathrm{e}^{-\mathbb{i}\theta}\,\breve{f^>}}{2}\,.$$

Moreover, $\forall\theta \in [0, 2\pi[$, a θ-symmetrical function f_1 and a $\theta+\pi$-symmetrical one f_2 are orthogonal:

$$\langle f_1 | f_2 \rangle = \langle f_1^< | f_2^< \rangle + \langle f_1^> | f_2^> \rangle = \langle \breve{f_1^>} | \overline{\mathrm{e}^{\mathbb{i}\theta}}\, \mathrm{e}^{\mathbb{i}(\theta+\pi)} | \breve{f_2^>} \rangle + \langle f_1^> | f_2^> \rangle = -\langle \breve{f_1^>} | \breve{f_2^>} \rangle + \langle f_1^> | f_2^> \rangle = 0\,.$$

3 Complex spectrum of the Coulomb potential

Contrary to $H_\omega(\mathbb{R}_+^*)$, H admits states of complex eigenvalue, included in $L^2(\mathbb{R}_+^*)$, as for example the following function in Fig. 1. This results from $m_\pm \neq 0$[3].

These solutions are however orthogonal to the quest of self-adjoint extensions and need not to by studied furthermore.

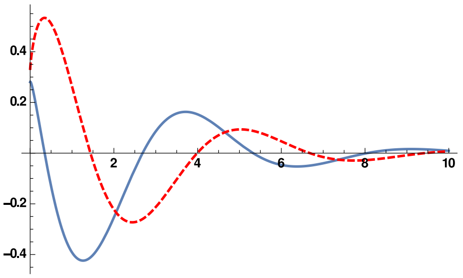

Figure 1: Curves of the real (plain line) and imaginary (dashed line) parts of a function ϕ, solution of $-\phi''(x) - \phi(x)/x = (1+\mathbb{i})\phi(x)$.

4 Self-adjoint extensions of $\mathbb{R}^{3*}$

.

If one defines eigenstates in $L^2(\mathbb{R}^{3*})$ (the pointed space, where the position of the fixed charged particle is excluded), one finds that the classification of $H(\mathbb{R}^{3*})$ extensions is equal to that of $H(\mathbb{R}_+^*)$ ones[2].

This exclusion results from the Coulomb potential emitted by the particle, but the other charged particle is then treated separately from the first one. If one makes the standard transformation of the two-particle problem into a virtual charge submitted to an effective Coulomb potential centered at the barycentre, the exclusion of the barycentre is not founded anymore.

Eventually, this choice would have no influence on the final discussion of physical states in three dimension, since, as explained in the article, only Rydberg states have ever been observed.

References

[1] Abramovici, G. & Avishai, Y. J. The one-dimensional Coulomb problem. *Phys. A: Math. Theor.* **42**, 285302 (2009).

[2] De Oliveira, C. R. & Verri, A. A. Self-adjoint extensions of Coulomb systems in 1, 2 and 3 dimensions. *Ann. Phys.* **324**, 251-266 (2009).

[3] Gitman, D. M., Tyutin, I. V. & Voronov, B. L. *Self-adjoint Extension in Quantum Mechanics.* (Birkhäuser, Boston, 2012).
